# Sucrose Abstinence and Environmental Enrichment Effects on Mesocorticolimbic DARPP32 in Rats

**DOI:** 10.1038/s41598-018-29625-x

**Published:** 2018-09-04

**Authors:** Jeffrey W. Grimm, Edwin Glueck, Darren Ginder, Jeff Hyde, Katherine North, Kyle Jiganti

**Affiliations:** Department of Psychology and Program in Behavioral Neuroscience, Western Washington University, Bellingham, Washington USA

## Abstract

Dopamine- and cAMP-regulated neuronal phosphoprotein 32 kDa (DARPP32) is a signaling molecule that could serve as a molecular switch, promoting or restraining sucrose seeking. We measured DARPP32 and pThr34 DARPP32 in the brains of male Long-Evans rats with a history of sucrose self-administration followed by 1 or 30 days of abstinence and exposure to either overnight (acute) or one month (chronic) environmental enrichment (EE). Brains were extracted following a 1 h cue reactivity test or no exposure to the test environment. Micropunches (prelimbic, infralimbic, and anterior cingulate areas of the medial prefrontal cortex, orbitofrontal cortex, dorsal striatum, nucleus accumbens, and ventral tegmental area) were then processed using Western blot. Abstinence increased, while EE decreased, sucrose seeking. DARPP32 and pThr34 DARPP32 levels were affected by testing, abstinence, and/or EE in most regions. Especially salient results were observed in the nucleus accumbens core, a region associated with relapse behaviors. Both acute and chronic EE reduced DARPP32 in the nucleus accumbens core and acute EE increased the ratio of phosphorylated to total DARPP32. Degree of DARPP32 phosphorylation negatively correlated with sucrose seeking. These findings demonstrate a potential role for DARPP32 in mediating the “anti-craving” effect of EE.

## Introduction

Dopamine- and cAMP-regulated neuronal phosphoprotein 32 kDa (DARPP32) is an intracellular protein identified as a key regulator of dopamine neurotransmission and dopamine-mediated behaviors^1^. Its activity in the nucleus accumbens is of particular interest in addiction neuroscience^2^.

While several neurotransmitters and their respective receptors indirectly regulate DARPP32, the effect of dopamine via D1 receptors may be most relevant to changes in synaptic plasticity endemic to addiction^2,3^. Specifically, dopamine D1 receptor agonism increases activity of protein kinase A which in turn promotes phosphorylation of DARPP32 at threonine 34. This phosphorylated DARPP32 (pThr34 DARPP32) then attenuates activity of protein phosphatase 1, thereby releasing its inhibition of the MEK/ERK pathway. This ultimately promotes CREB activation and subsequent gene expression including expression of immediate-early genes such as c-fos^4^.

Dopamine D1 receptors are particularly important in two phenomena that may reflect changes in motivation to seek sucrose by rats, an animal model used to better understand food and drug addiction behaviors, behaviors with significant neurobehavioral overlap^5^. First, D1 receptors are involved in the abstinence-dependent increase (incubation) in sucrose seeking. Blocking D1 receptors is more effective at reducing sucrose seeking after one day versus one month of abstinence^6^. Second, sucrose seeking markedly reduced by exposure to environmental enrichment (EE) was restored with a D1 agonist^7^.

**Figure 1:**
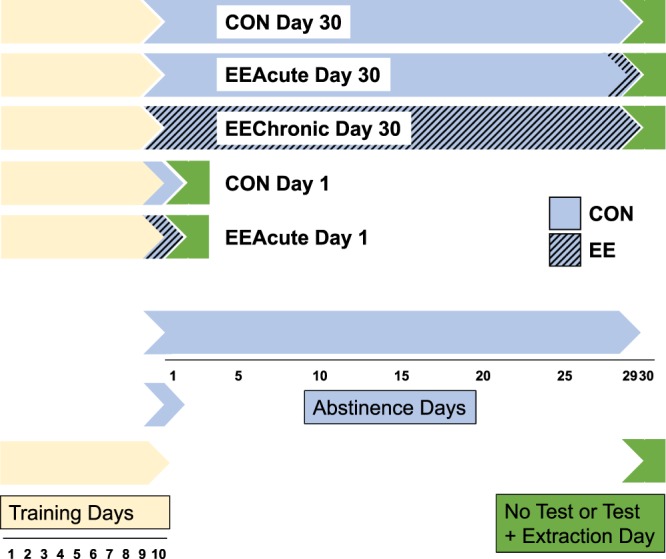
Experimental timeline. All subjects received the same Training. After the final Training session, subjects were pseudo-randomly assigned to the various treatment conditions that varied by length of abstinence, exposure to EE, and whether subjects would be tested (allowed to respond for sucrose-paired cues) prior to brain extraction.

These changes in sensitivity to D1 antagonist and agonist could relate to altered intracellular signaling via DARPP32. Therefore, in the present study we sought to determine whether measures of DARPP32, pThr34 DARPP32, and the ratio of the measures would vary with increased sucrose seeking following incubation and/or decreased sucrose seeking following exposure to EE. The experimental timeline is presented in Fig. 1. Briefly, adult, male Long-Evans rats first self-administered sucrose in 10 daily 2-h sessions, and then some were reintroduced to the self-administration environment prior to brain extraction (TEST vs. NO TEST indicated in Tables and Figures). Housing and length of abstinence were also manipulated with rats experiencing 1 or 29 days of abstinence from sucrose and either control, overnight EE, or 29 days of EE. These conditions are indicated as CON, EEAcute, and EEChronic, respectively, in text, Tables, and Figures. Nucleus accumbens core and shell sub-regions were examined, as were seven other brain regions including regions of medial prefrontal cortex (mPFC), orbitofrontal cortex, striatum, and midbrain.

## Results

In most instances only statistics for significant main effects and interactions of ANOVA are noted in the text. Means ± standard error of the mean (SEM) are indicated in the text, tables, and on the Figures. Ten subjects were removed from the study for failure to acquire reliable self-administration (at least 20 sucrose deliveries per day for the final 3 days of Training) and 6 brains were damaged in processing leaving 126 subjects for analyses.

### Body weight

Average weights did not differ between treatment conditions prior to the start of the study. Weights in grams were 414.0 ± 3.0. As reported previously^7,8^, chronic EE did not affect body weight (*t*(48) = 1.0, *p* = 0.2). Final weights in grams for rats in the Day 30 CON and EEChronic conditions were 470.0 ± 7.2 and 459.4 ± 7.2, respectively.

### Behavioral procedures

#### Training

**Figure 2:**
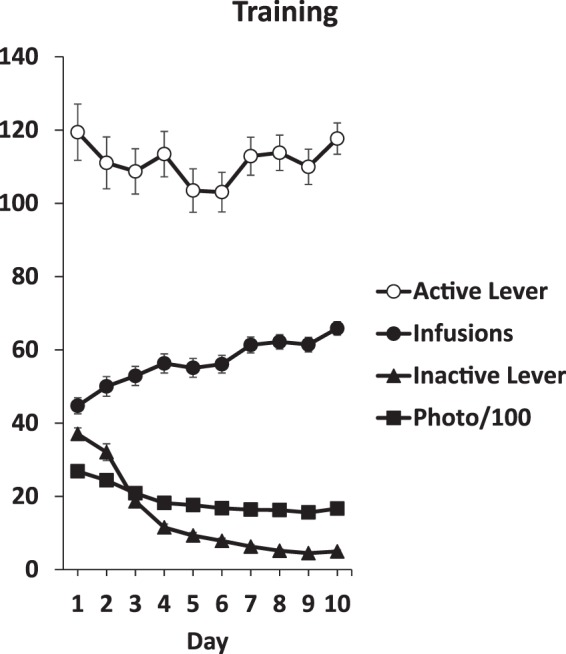
Training data. Data are collapsed across experimental conditions as there were no group differences (see Results). Depicted are active and inactive lever responses, infusions (sucrose deliveries), and photobeam breaks per daily 2-h session.

There were no significant differences between groups in Training measures. TIME was significant for active lever responses, *F*(4,464) = 3.2, *p* < 0.05, infusions *F*(4,464) = 6.9, *p* < 0.001, and inactive lever responses, *F*(4,464) = 14.5, *p* < 0.001. Active lever responding and infusions increased, while inactive lever responding decreased, over the 5 final days of training. The mean ± SEM of each of these measures on the tenth day of Training were active responses 119.1 ± 4.5, infusions 65.7 ± 1.8, inactive responses 5.0 ± 0.4, and photobeam breaks 1666.0 ± 48.8. All Training data, collapsed across experimental conditions, are presented in Fig. 2.

#### Testing

**Figure 3:**
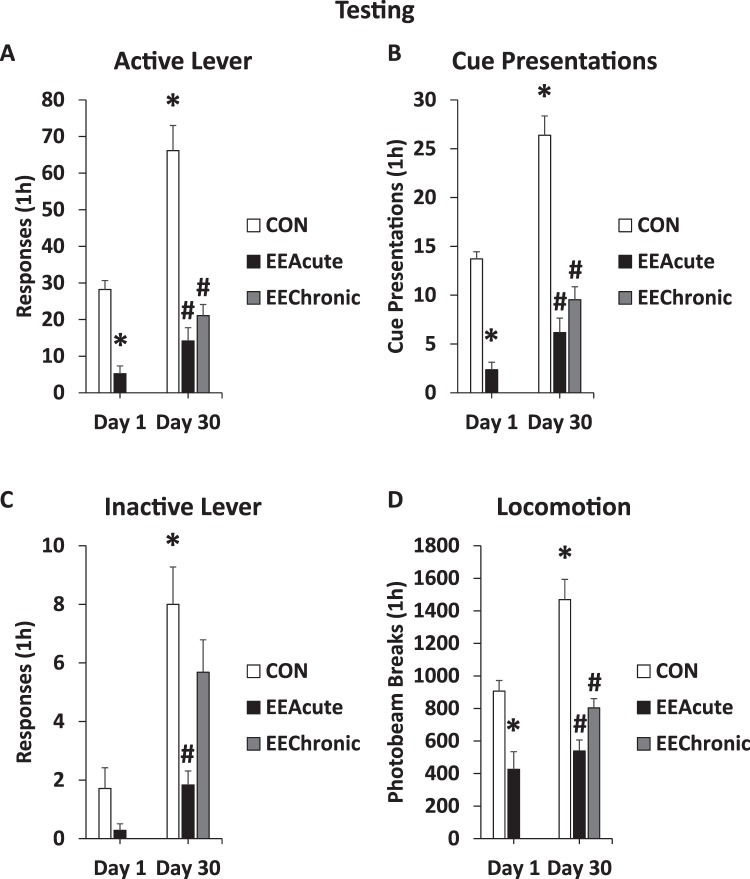
Testing behaviors. Tested rats were allowed to respond for the sucrose-paired tone + light cue for 1 h prior to brain extraction. *indicates statistically significant from CON Day 1, *p* < 0.05. ^#^indicates statistically significant from CON Day 30, *p* < 0.05. As indicated, panel (A) indicates active lever reponses, (**B)** cue presentations, (**C**) inactive lever responses, and (**D**), photobeam breaks as a measure of locomotion.

Post-hoc results are indicated on Fig. 3. Rats responded more on the active lever after 30 days of abstinence, and responding was decreased by either acute or chronic EE (Fig. 3A). For active lever responding there were significant effects of DAY (length of abstinence) *F*(1,61) = 35.7, *p* < 0.001 and HOUSING (CON, EEAcute, EEChronic) *F*(2,61) = 50.1, *p* < 0.001. There was also a significant interaction of DAY X HOUSING *F*(1,61) = 13.7, *p* < 0.001. For infusions (cue presentations, as sucrose was not available; Fig. 3B) there were significant effects of DAY *F*(1,61) = 39.8, *p* < 0.001 and HOUSING *F*(2,61) = 66.3, *p* < 0.001, and a significant interaction of DAY X HOUSING *F*(1,61) = 11.5, *p* < 0.01. For inactive lever responding (Fig. 3C) there were main effects of DAY *F*(1,61) = 21.5, *p* < 0.001 and HOUSING *F*(2,61) = 12.8, *p* < 0.001. There was also a significant interaction between DAY X HOUSING *F*(1,61) = 7.8, *p* < 0.01. For photobeam breaks (Fig. 3D) there were also significant effects of DAY *F*(1,61) = 14.1, *p* < 0.001 and HOUSING *F*(2,61) = 27.4, *p* < 0.001, as well as a significant interaction between DAY X HOUSING *F*(1,61) = 6.3, *p* < 0.05.

### Western blot

Table 1 presents not tested control Day 1 image intensity values across all brain regions examined. Ratio indicates the ratio of pThr34 DARPP32 to DARPP32. One observation is that DARPP32 signal, and to a lesser extent pThr34 DARPP32 signal, was highest in dorsal and ventral (accumbens) striatal regions. Another is that, in the VTA, the intensity of pThr34 DARPP32 was relatively greater than that for DARPP32. This finding is not due to a loss of potency of DARPP32 antibody as the samples for DMS and DLS were run over the succeeding several weeks with aliquots of the same lot. Those intensity values were higher and similar to values for ventral striatal and anterior cortex run many weeks earlier. More likely “total” DARPP32 and pThr34 DARPP32 antibodies recognize different forms of the DARPP32 protein. This conclusion is supported by the migrations of total and phosphorylated DARPP32 (Supplement 1). The pThr34 DARPP32 is hyper-shifted, migrating slower than the DARPP32, possibly due to an effect of phosphorylation on the conformation and/or charge on the protein. Therefore, one needs to be cautious regarding interpreting the Ratio as an absolute indication of percent of total DARPP32 that is in the pThr34 phosphorylated state.

**Table 1:** Intensity and Ratio values for NO TEST CON Day 1 group across brain regions.

Region	DARPP32	pThr34 DARPP32	Ratio
Prelimbic mPFC (12)	0.59 ± 0.06	0.29 ± 0.04	0.49 ± 0.04
Orbitofrontal cortex (12)	0.89 ± 0.11	0.33 ± 0.06	0.37 ± 0.05
Infralimbic mPFC (12)	0.61 ± 0.03	0.30 ± 0.03	0.49 ± 0.04
Anterior cingulate mPFC (12)	0.60 ± 0.05	0.22 ± 0.03	0.41 ± 0.05
Dorsomedial striatum (11)	1.28 ± 0.08	0.47 ± 0.03	0.38 ± 0.02
Dorsolateral striatum (12)	2.48 ± 0.25	0.57 ± 0.06	0.27 ± 0.04
Nucleus accumbens core (12)	1.96 ± 0.12	0.40 ± 0.04	0.20 ± 0.02
Nucleus accumbens shell (10)	1.46 ± 0.13	0.35 ± 0.05	0.25 ± 0.04
Ventral tegmental area (10)	0.19 ± 0.03	0.31 ± 0.05	1.83 ± 0.31

Note: “Ratio” is the ratio of pThr34 DARPP32 to DARPP32. Means ± SEMs are indicated. N sizes are in parentheses.

**Figure 4:**
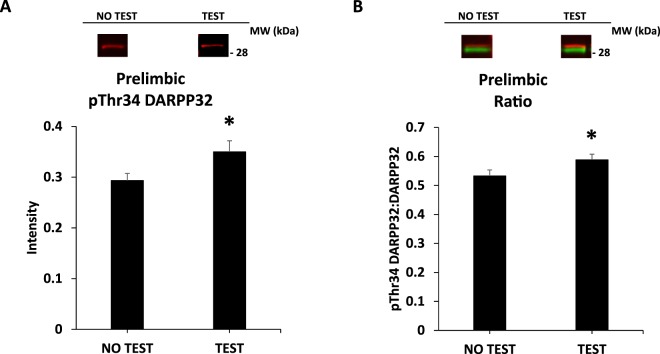
DARPP32 in the prelimbic area of the mPFC. Panel (A) depicts pThr34 DARPP32 alone and (**B**), the phosphorylation of DARPP32 at Thr34 as a ratio value. *indicates statistically significant from NO TEST, *p* < 0.05. Representative blots for each condition are provided.

**Figure 5:**
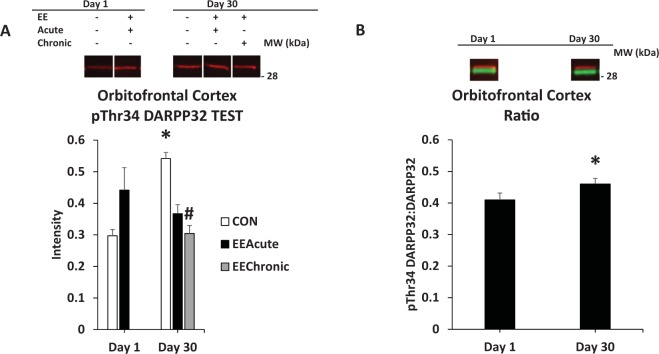
DARPP32 in the OFC. Panel (A) depicts pThr34 DARPP32 in tested subjects across all other experimental conditions; (**B**) depicts phosphorylation of DARPP32 at Thr34 as a ratio value for subjects comparing Days 1 and 30 of abstinence. *indicates statistically different from Day 1 condition, *p* < 0.05. ^#^indicates statistically significant from CON Day 30, *p* < 0.05. Representative blots for each condition are provided.

**Figure 6:**
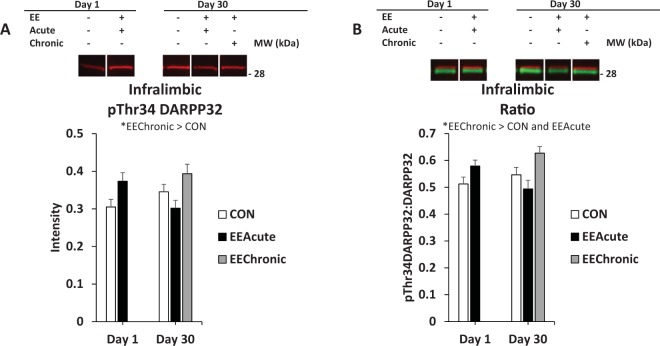
DARPP32 in infralimbic area of mPFC. Panel (A) depicts pThr34 in all rats (not-tested and tested) in all experimental conditions; panel (B) depicts phosphorylation of DARPP32 at Thr34 as a ratio value, also in all rats (not-tested and tested) in all experimental conditions. *indicates statistically significant difference(s), *p* < 0.05. Representative blots for each condition are provided.

**Figure 7:**
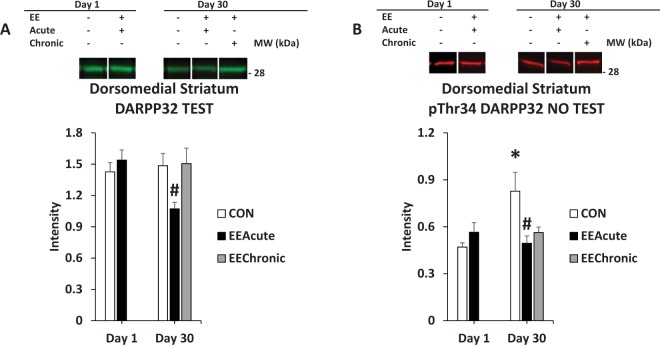
DARPP32 in DMS. Panel (A) depicts DARPP32 in tested subjects in all experimental conditions; panel (B) depicts phosphorylation of DARPP32 at Thr34 as a ratio value in rats that were not tested, but also in all experimental conditions. *indicates statistically significant from CON Day 1, *p* < 0.05. ^#^indicates statistically significant from CON Day 30, p < 0.05. Representative blots for each condition are provided.

**Figure 8:**
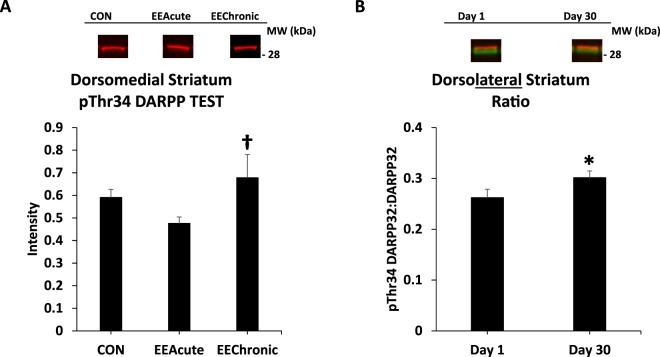
DARPP32 in DMS (cont.) and in DLS. Panel (A) depicts pThr34 DARPP32 in all subjects across housing conditions; Panel (B) depicts phosphorylation of DARPP32 at Thr34 as a ratio value in all rats comparing Days 1 and 30 of abstinence. *indicates statistically significant from Day 1, *p* < 0.05. ^†^indicates statistically significant from EEAcute, *p* < 0.05. Representative blots for each condition are provided.

**Figure 9:**
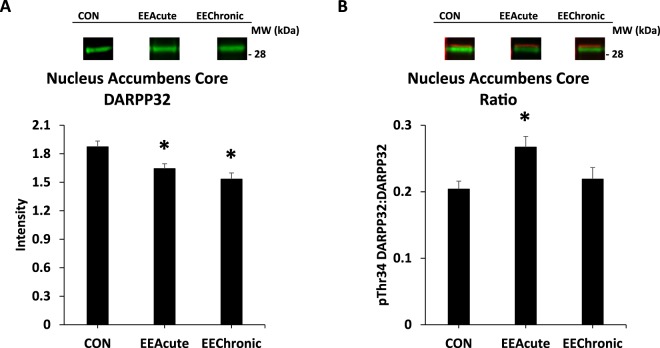
DARPP32 in nucleus accumbens core. Panel (A) depicts DARPP32 in all subjects in all housing conditions; panel (B) represents these same groupings, but depicts phosphorylation of DARPP32 at Thr34 as a ratio value. *indicates statistically significant from CON, *p* < 0.05. Representative blots for each condition are provided.

**Figure 10:**
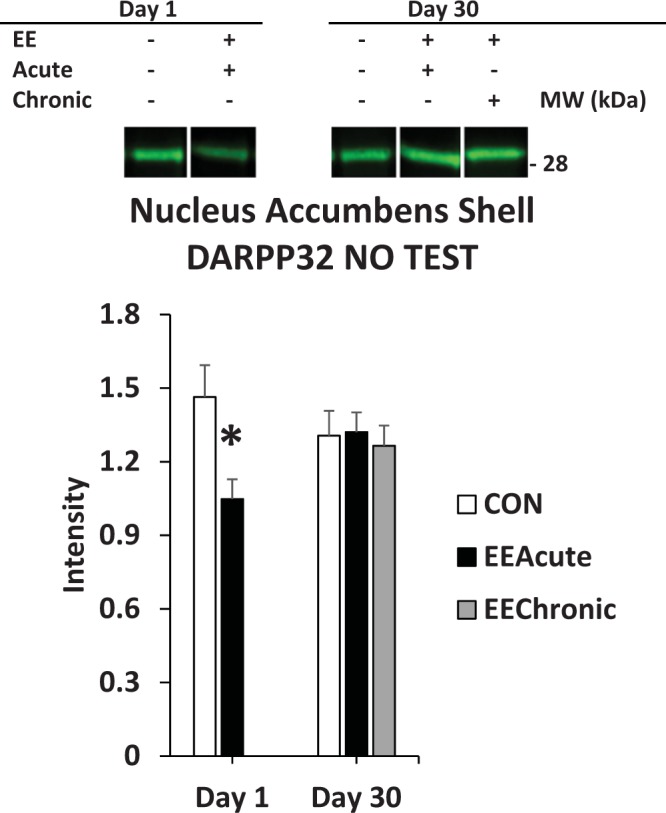
DARPP32 in nucleus accumbens shell. DARPP32 in subjects that were not tested, but in all other experimental conditions. *indicates statistically significant from CON Day 1, *p* < 0.05. Representative blots for each condition are provided.

**Figure 11:**
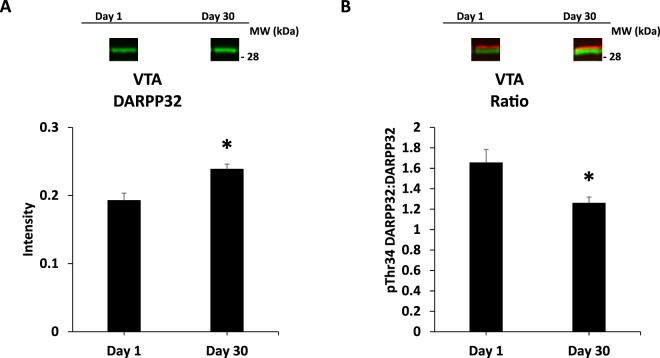
DARPP32 in VTA. Panel (A) depicts DARPP32 in all subjects, comparing Days 1 and 30 of abstinence; Panel (B) compares phosphorylation of DARPP32 at Thr34 as a ratio value on Days 1 and 30 of abstinence. *indicates statistically significant from Day 1, *p* < 0.05. Representative blots for each condition are provided.

ANOVA results for all conditions by brain region (rostral to caudal) are presented below. Only statistically significant effects and/or interactions are indicated. Degrees of freedom values vary across brain regions as availability of samples (tissue collection problems, low protein in samples) varied across brain regions. *All data*, grouped by treatment condition, are presented in Tables 2–6. *Only statistically significant* main effects or interactions following ANOVA findings are indicated in Figs 4–11.

#### Prelimbic area of the mPFC

There were main effects of TEST for pThr34 DARPP32, TEST *F*(1,115) = 4.7, *p* < 0.05, and for Ratio, TEST *F*(1,115) = 4.4, *p* < 0.05 (Table 2 and Fig. 4).

**Table 2:** Prelimbic mPFC and Orbitofrontal cortex. Intensity and Ratio values across brain regions and conditions.

	CON Day 1	EEAcute Day 1	CON Day 30	EEAcute Day 30	EEChronic Day 30
Prelimbic NO TEST	(12)	(12)	(11)	(12)	(12)
DARPP32	0.59 ± 0.06	0.51 ± 0.04	0.67 ± 0.06	0.53 ± 0.05	0.60 ± 0.07
pThr34 DARPP32	0.29 ± 0.04	0.32 ± 0.04	0.29 ± 0.02	0.27 ± 0.03	0.30 ± 0.03
Ratio	0.49 ± 0.04	0.64 ± 0.07	0.45 ± 0.03	0.54 ± 0.04	0.54 ± 0.04
Prelimbic TEST	(14)	(14)	(13)	(13)	(12)
DARPP32	0.56 ± 0.06	0.49 ± 0.02	0.68 ± 0.12	0.69 ± 0.12	0.61 ± 0.05
pThr34 DARPP32	0.31 ± 0.04	0.31 ± 0.02	0.41 ± 0.06	0.38 ± 0.07	0.35 ± 0.02
Ratio	0.53 ± 0.04	0.60 ± 0.04	0.65 ± 0.03	0.55 ± 0.05	0.62 ± 0.06
Orbitofrontal NO TEST	(12)	(12)	(11)	(12)	(12)
DARPP32	0.89 ± 0.11	0.74 ± 0.06	0.82 ± 0.06	0.82 ± 0.07	1.03 ± 0.09
pThr34 DARPP32	0.33 ± 0.06	0.31 ± 0.03	0.32 ± 0.02	0.40 ± 0.03	0.47 ± 0.06
Ratio	0.37 ± 0.05	0.43 ± 0.02	0.41 ± 0.03	0.51 ± 0.05	0.44 ± 0.03
Orbitofrontal TEST	(13)	(13)	(14)	(12)	(13)
DARPP32	0.73 ± 0.03	0.91 ± 0.07	1.13 ± 0.18	0.79 ± 0.06	0.85 ± 0.09
pThr34 DARPP32	0.30 ± 0.03	0.44 ± 0.07	0.54 ± 0.07	0.37 ± 0.03	0.30 ± 0.03
Ratio	0.41 ± 0.05	0.43 ± 0.05	0.51 ± 0.06	0.47 ± 0.04	0.40 ± 0.04

Note: “Ratio” is the ratio of pThr34 DARPP32 to DARPP32. Means ± SEMs are indicated. N sizes are in parentheses.

#### Orbitofrontal cortex (OFC)

For DARPP32 there were two significant interactions: TEST X HOUSING *F*(2,114) = 3.3, *p* < 0.05 and DAY X HOUSING *F*(1,114) = 5.8, *p* < 0.05. This prompted ANOVA of NO TEST and TEST conditions. For TEST subjects, there was a significant interaction, DAY X HOUSING *F*(1,60) = 5.9, *p* < 0.05. For Thr34 DARPP32 there were also two significant interactions: TEST X HOUSING *F*(2,114) = 8.0, *p* < 0.01, and TEST X DAY *F*(1,114) = 8.9, *p* < 0.01. Subsequent ANOVAs revealed, for TEST subjects, an effect of HOUSING *F*(2,60) = 5.7, *p* < 0.01 and a significant interaction of DAY X HOUSING *F*(1,60) = 9.1, *p* < 0.01 (Table 2 and Fig. 5).

#### Infralimbic area of the mPFC

For pThr34 DARPP32 there was a main effect of HOUSING *F*(2,114) = 4.5, *p* < 0.05, and a significant interaction, DAY X HOUSING *F*(1,114) = 6.3, *p* < 0.05. For Ratio there was a significant effect of HOUSING *F*(2,114) = 6.7, *p* < 0.01 and a significant interaction, DAY X HOUSING *F*(1,114) = 5.0, *p* < 0.05. No post-hoc tests following the interactions were statistically significant (Table 3 and Fig. 6).

**Table 3:** Infralimbic and Anterior Cingulate mPFC. Intensity and Ratio values across brain regions and conditions.

	CON Day 1	EEAcute Day 1	CON Day 30	EEAcute Day 30	EEChronic Day 30
Infralimbic NO TEST	(12)	(12)	(11)	(12)	(12)
DARPP32	0.61 ± 0.03	0.62 ± 0.04	0.67 ± 0.05	0.57 ± 0.03	0.64 ± 0.05
pThr34 DARPP32	0.30 ± 0.03	0.36 ± 0.03	0.32 ± 0.03	0.28 ± 0.03	0.43 ± 0.04
Ratio	0.49 ± 0.04	0.57 ± 0.03	0.49 ± 0.04	0.48 ± 0.05	0.67 ± 0.03
Infralimbic TEST	(14)	(14)	(12)	(12)	(13)
DARPP32	0.56 ± 0.04	0.65 ± 0.06	0.60 ± 0.03	0.69 ± 0.05	0.62 ± 0.04
pThr34 DARPP32	0.31 ± 0.03	0.38 ± 0.04	0.37 ± 0.02	0.32 ± 0.03	0.36 ± 0.03
Ratio	0.53 ± 0.03	0.59 ± 0.03	0.60 ± 0.03	0.51 ± 0.04	0.59 ± 0.03
Ant. Cingulate NO TEST	(12)	(12)	(12)	(12)	(11)
DARPP32	0.60 ± 0.05	0.61 ± 0.03	0.56 ± 0.03	0.64 ± 0.05	0.58 ± 0.04
pThr34 DARPP32	0.22 ± 0.03	0.21 ± 0.02	0.17 ± 0.03	0.22 ± 0.02	0.24 ± 0.03
Ratio	0.41 ± 0.05	0.35 ± 0.04	0.33 ± 0.06	0.35 ± 0.04	0.42 ± 0.06
Ant. Cingulate TEST	(14)	(14)	(12)	(12)	(13)
DARPP32	0.60 ± 0.05	0.66 ± 0.05	0.56 ± 0.04	0.58 ± 0.04	0.57 ± 0.03
pThr34 DARPP32	0.18 ± 0.02	0.19 ± 0.03	0.15 ± 0.03	0.21 ± 0.02	0.17 ± 0.03
Ratio	0.33 ± 0.04	0.30 ± 0.04	0.27 ± 0.06	0.38 ± 0.03	0.33 ± 0.07

Note: “Ratio” is the ratio of pThr34 DARPP32 to DARPP32. Means ± SEMs are indicated. N sizes are in parentheses.

#### Anterior cingulate cortex

No significant effects and/or interactions were observed (Table 3).

#### Dorsomedial striatum (DMS)

For DARPP32 there was a significant effect of HOUSING *F*(2,113) = 5.0, *p* < 0.01, and two significant interactions: TEST X DAY *F*(1,113) = 7.2, *p* < 0.01 and DAY X HOUSING *F*(1,113) = 4.8, *p* < 0.05. Subsequent ANOVAs revealed for TEST subjects a significant effect of HOUSING *F*(2,59) = 4.9, *p* < 0.05 and a significant interaction of DAY X HOUSING *F*(1,59) = 5.8, *p* < 0.05. For pThr34 DARPP32 there was also a significant effect of HOUSING *F*(2,113) = 7.2, *p* < 0.01 and two significant interactions: TEST X HOUSING *F*(2,113) = 3.3, *p* < 0.05 and DAY X HOUSING *F*(1,113) = 7.8, *p* < 0.01. Follow-up ANOVAs revealed for NO TEST subjects significant effects of DAY *F*(1,54) = 4.4, *p* < 0.05, HOUSING *F*(2,54) = 6.7, *p* < 0.01, and a significant interaction of DAY X HOUSING *F*(1,54) = 9.8, *p* < 0.01. For TEST subjects, there was a significant effect of HOUSING *F*(2,59) = 3.4, *p* < 0.05. For Ratio there was a significant interaction of TEST X DAY *F*(1,113) = 8.2, *p* < 0.01. Subsequent ANOVAs revealed a significant interaction of DAY X HOUSING *F*(1,54) = 8.0, *p* < 0.01 for the NO TEST subjects (Table 4 and Figs 7 and 8).

**Table 4:** Dorsal Striatal regions. Intensity and Ratio values across brain regions and conditions.

	CON Day 1	EEAcute Day 1	CON Day 30	EEAcute Day 30	EEChronic Day 30
Dorsomedial NO TEST	(11)	(12)	(12)	(12)	(12)
DARPP32	1.28 ± 0.08	1.15 ± 0.09	1.58 ± 0.17	1.29 ± 0.10	1.26 ± 0.08
pThr34 DARPP32	0.47 ± 0.03	0.57 ± 0.06	0.83 ± 0.12	0.49 ± 0.05	0.56 ± 0.04
Ratio	0.38 ± 0.02	0.50 ± 0.04	0.56 ± 0.08	0.41 ± 0.04	0.47 ± 0.03
Dorsomedial TEST	(13)	(13)	(13)	(12)	(13)
DARPP32	1.43 ± 0.09	1.54 ± 0.10	1.48 ± 0.12	1.07 ± 0.06	1.51 ± 0.15
pThr34 DARPP32	0.57 ± 0.05	0.50 ± 0.04	0.61 ± 0.05	0.45 ± 0.04	0.68 ± 0.10
Ratio	0.40 ± 0.03	0.34 ± 0.03	0.43 ± 0.03	0.44 ± 0.03	0.45 ± 0.05
Dorsolateral NO TEST	(12)	(12)	(12)	(12)	(12)
DARPP32	2.48 ± 0.25	2.22 ± 0.18	2.05 ± 0.13	1.92 ± 0.09	2.27 ± 0.12
pThr34 DARPP32	0.57 ± 0.06	0.48 ± 0.05	0.66 ± 0.09	0.60 ± 0.05	0.56 ± 0.04
Ratio	0.27 ± 0.04	0.23 ± 0.03	0.32 ± 0.05	0.32 ± 0.02	0.26 ± 0.02
Dorsolateral TEST	(14)	(14)	(13)	(12)	(13)
DARPP32	2.07 ± 0.16	2.00 ± 0.15	1.86 ± 0.12	2.17 ± 0.24	2.19 ± 0.13
pThr34 DARPP32	0.57 ± 0.07	0.50 ± 0.07	0.62 ± 0.06	0.56 ± 0.06	0.64 ± 0.07
Ratio	0.29 ± 0.03	0.26 ± 0.03	0.34 ± 0.03	0.28 ± 0.04	0.30 ± 0.03

Note: “Ratio” is the ratio of pThr34 DARPP32 to DARPP32. Means ± SEMs are indicated. N sizes are in parentheses.

#### Dorsolateral striatum (DLS)

For Ratio there was a significant effect of DAY *F*(1,116) = 4.9, *p* < 0.05 (Table 4 and Fig. 8).

#### Nucleus accumbens core

For DARPP32 there was a significant effect of HOUSING *F*(2,112) = 4.4, *p* < 0.05. For Ratio there was also a significant effect of HOUSING *F*(2,112) = 3.1, *p* < 0.05 (Table 5 and Fig. 9).

**Table 5:** Ventral Striatal regions. Intensity and Ratio values across brain regions and conditions.

	CON Day 1	EEAcute Day 1	CON Day 30	EEAcute Day 30	EEChronic Day 30
N. Acc. Core NO TEST	(12)	(12)	(11)	(12)	(12)
DARPP32	1.96 ± 0.12	1.69 ± 0.13	1.88 ± 0.09	1.72 ± 0.08	1.49 ± 0.08
pThr34 DARPP32	0.40 ± 0.04	0.36 ± 0.04	0.37 ± 0.05	0.40 ± 0.04	0.36 ± 0.04
Ratio	0.20 ± 0.02	0.23 ± 0.03	0.20 ± 0.03	0.26 ± 0.04	0.24 ± 0.02
N. Acc. Core TEST	(13)	(14)	(11)	(12)	(13)
DARPP32	1.80 ± 0.16	1.51 ± 0.08	1.85 ± 0.11	1.67 ± 0.11	1.58 ± 0.11
pThr34 DARPP32	0.43 ± 0.05	0.44 ± 0.05	0.32 ± 0.05	0.47 ± 0.06	0.31 ± 0.04
Ratio	0.23 ± 0.02	0.31 ± 0.04	0.18 ± 0.02	0.27 ± 0.02	0.20 ± 0.03
N. Acc. Shell NO TEST	(10)	(11)	(12)	(13)	(12)
DARPP32	1.46 ± 0.13	1.05 ± 0.08	1.31 ± 0.10	1.32 ± 0.08	1.27 ± 0.08
pThr34 DARPP32	0.35 ± 0.05	0.35 ± 0.03	0.32 ± 0.01	0.38 ± 0.03	0.38 ± 0.02
Ratio	0.25 ± 0.04	0.36 ± 0.05	0.27 ± 0.03	0.30 ± 0.03	0.32 ± 0.03
N. Acc. Shell TEST	(14)	(14)	(12)	(11)	(12)
DARPP32	1.23 ± 0.08	1.35 ± 0.13	1.19 ± 0.08	1.10 ± 0.10	1.11 ± 0.05
pThr34 DARPP32	0.36 ± 0.04	0.31 ± 0.02	0.34 ± 0.03	0.32 ± 0.03	0.35 ± 0.01
Ratio	0.29 ± 0.02	0.25 ± 0.03	0.28 ± 0.03	0.30 ± 0.03	0.32 ± 0.02

Note: “Ratio” is the ratio of pThr34 DARPP32 to DARPP32. Means ± SEMs are indicated. N sizes are in parentheses.

#### Nucleus accumbens shell

For DARPP32 there was a significant interaction of TEST X DAY *F*(1,111) = 5.7, *p* < 0.05. Subsequent ANOVAs revealed for the NO TEST subjects a significant interaction of DAY X HOUSING *F*(1,53) = 5.1, *p* < 0.05 (Table 5 and Fig. 10).

#### Ventral tegmental area (VTA)

For DARPP32 there was a significant effect of DAY *F*(1,113) = 14.4, *p* < 0.001. For pThr34 DARPP32 there was a significant interaction of TEST X DAY *F*(1,113) = 4.0, *p* < 0.05. Follow-up ANOVAs did not identify any significant effects or interactions. For Ratio there was a significant effect of Day *F*(1,113) = 9.0, *p* < 0.01 (Table 6 and Fig. 11).

**Table 6:** VTA. Intensity and Ratio values across conditions.

	CON Day 1	EEAcute Day 1	CON Day 30	EEAcute Day 30	EEChronic Day 30
VTA NO TEST	(10)	(12)	(13)	(11)	(12)
DARPP32	0.19 ± 0.03	0.19 ± 0.02	0.27 ± 0.02	0.25 ± 0.02	0.19 ± 0.02
pThr34 DARPP32	0.31 ± 0.05	0.25 ± 0.03	0.39 ± 0.03	0.33 ± 0.04	0.27 ± 0.04
Ratio	1.83 ± 0.31	1.35 ± 0.11	1.58 ± 0.20	1.23 ± 0.09	1.34 ± 0.19
VTA TEST	(14)	(13)	(13)	(12)	(13)
DARPP32	0.20 ± 0.02	0.19 ± 0.02	0.25 ± 0.02	0.22 ± 0.01	0.25 ± 0.02
pThr34 DARPP32	0.30 ± 0.03	0.29 ± 0.03	0.30 ± 0.04	0.23 ± 0.03	0.33 ± 0.04
Ratio	1.56 ± 0.16	1.91 ± 0.38	1.16 ± 0.09	0.97 ± 0.12	1.25 ± 0.11

Note: “Ratio” is the ratio of pThr34 DARPP32 to DARPP32. Means ± SEMs are indicated. N sizes are in parentheses.

#### Correlations

Significant correlations between protein image intensity and active lever responding were found only in the nucleus accumbens core. Active lever responding during the Testing session was negatively correlated with pThr34 DARPP32 intensity, *r* = −0.4, *p* < 0.01, and with the ratio of pThr34 DARPP32 to total DARPP32, *r* = −0.5, p < 0.001.

## Discussion

As observed previously (e.g.)^9^, sucrose seeking was greater after 30 versus 1 day of abstinence (incubation of sucrose seeking). In addition, either acute or chronic EE was effective at reducing sucrose seeking^10^. DARPP32 and pThr34 DARPP32 levels were associated with the abstinence and EE manipulations; effects varied by brain region examined.

Examples of incubation-specific effects were in the OFC, DMS, DLS, and VTA. In the OFC, in tested subjects, pThr34 DARPP32 signal and the Ratio value was greater on Day 30 versus Day 1 for control subjects (Fig. 5). These effects are especially salient as this brain region has a role in reward valuation^11^. pThr34 phosphorylation varied with duration of abstinence, suggesting a role for the region in identification of a change in reward salience in abstinence.

The incubation-related effects in the DMS and DLS are intriguing as these brain regions, especially the DLS, have been implicated in the transition from goal-directed to habit-based responding^12^. In the DMS subjects that were not tested, there was an abstinence-dependent increase in pThr34 DARPP32 signal whereas in the DLS there was an abstinence-dependent increase in the Ratio value regardless of whether rats were tested or not (Figs 7 and 8). As the DMS has also been associated with spatial learning^13^, it is notable that an incubation effect was observed in rats that had not been returned to the Testing environment. It could be that returning to the context of the test environment actually reversed this effect, perhaps due to dephosphorylation at pThr34 mediated by glutamate^4^. For the DLS, the abstinence-dependent increase in the Ratio value may reflect an increase in D1 signaling, the increased activity within DLS reflecting the greater responding of rats for the sucrose-paired cue. Essentially the rats are responding in extinction conditions, and compared to Day 1 of abstinence, the responding on Day 30 is more vigorous even in the face of non-reinforcement (habit).

In the VTA, DARPP32 signal was greater at Day 30 versus Day 1, and the Ratio value was greater at Day 1 versus Day 30 (Fig. 11). As the VTA is the source of dopamine innervating cortical and limbic structures, the changes in signaling here could have widespread impact. For example, the incubation effects in the VTA may have influenced the incubation effects described above for OFC, DMS, and DLS.

At this time there is very little in the literature to compare our incubation-specific results with. Abdolahi *et al*. examined the relationship between DARPP32 phosphorylation and incubation of nicotine seeking in rats^14^. There were incubation-related changes in the nucleus accumbens core, a decrease in pThr75 DARPP32, and an incubation of pThr34 DARPP32 in the insular cortex. It is difficult to compare between the present results and this other study, however, as the reinforcers are very different and the abstinence duration in the Abdolahi study was 7 days.

There were several EE-specific effects. In the infralimbic cortex, chronic EE increased pThr34 DARPP32 signal and the Ratio value (Fig. 6). While the exact role of infralimbic cortex in reward seeking is debated, it appears to have an important function in the acquisition of extinction learning^15^. “Activation” of this region by EE (e.g. observed by an increase in phosphorylation of DARPP32) could be indicative of the diminished responding by subjects following EE. This hypothesis is complicated by the lack of effect in the EEAcute Day 30 condition, and presupposes EE enhances extinction learning. Further study is required to explore the potential role of the infralimibc cortex in the anti-seeking effect of EE.

In the DMS pThr34 DARPP32 signal was greater in EEChronic versus EEAcute subjects, but only in the subjects that had been allowed to respond for sucrose-paired cues (Fig. 8). As noted above, there are previous findings supporting a role for the DMS in spatial learning^13^, and here again is an effect in the DMS that depends on exposure to the test environment. There are also findings indicating that the DMS contributes to the development of habit^16^. One possibility is that the DMS is involved in context-dependent expression of habit.

In the nucleus accumbens core both EEAcute and EEChronic DARPP32 signals were reduced versus controls. For Ratio, EEAcute signal was greater than control signal (both Fig. 9). These effects are, arguably, the most striking of the overall study. This is because it has been speculated that EE effects relate to changes in the motivational state of the subject^8^. Although novelty and housing-related stress may contribute, our working hypothesis based upon findings thus far is that the EE environment produces a behavioral contrast^17^ such that the Testing environment now has less incentive value (see^8,10^). This is reflected by diminished responding for reward-paired cues and, as we have found repeatedly, less motor activity which could indicate less motivation to explore. Within this framework, changes in DARPP32 availability and its phosphorylation indicate that EE impacts a key dopamine-mediated signal cascade protein in the nucleus accumbens core, a brain region repeatedly shown to be critical in attributing incentive salience to primary and secondary reinforcers^18^. The nucleus accumbens core was also the only brain region examined where correlations were found between protein measures and sucrose seeking behavior. We found negative correlations where decreased responding was associated with more pThr34 DARPP32 (either pThr34 DARPP32 or the Ratio value). Both the experimental and correlational findings therefore connect DARPP32 phosphorylation state in the nucleus accumbens core with the decreased sucrose seeking observed following EE.

EE effects that varied by abstinence were observed in the OFC, DMS, and nucleus accumbens shell. In the OFC, chronic EE decreased pThr32 DARPP32 signal (Fig. 5). The effects of EE in OFC again may relate to the importance of this structure in attributing value to stimuli. The fact that the change, at least in the EEChronic condition, was only in subjects that were allowed to respond for sucrose-paired cues could indicate that the OFC was processing the value of stimuli in the Testing context.

In the DMS, DARPP32 signal in subjects that were tested was decreased in EEAcute Day 30 subjects compared to control Day 30 subjects (Fig. 7A). In contrast, in subjects not tested, it was DMS pThr34 DARPP32 signal that was decreased (Fig. 7B). Although the magnitude of EE effects on sucrose seeking are relatively similar for early and late abstinence, these interactions between abstinence and EE in protein measures in the DMS could reflect the nature of abstinence-dependent processing of the return to the Testing context. For example, the blunting of the expression of associative learning by EE could be more or less dependent on DMS dopamine, depending on length of abstinence.

In the nucleus accumbens shell, acute EE reduced DARPP32 signal on Day 1, but not Day 30, only in subjects not re-exposed to the sucrose taking environment (Fig. 10). This was the only effect in the nucleus accumbens shell in the overall study. This was surprising, given the well-established role of this brain region in motivated behavior^19^. This particular effect, however, does identify a context-dependent effect of DARPP32 regulation. The shell is particularly important in tracking the outcome value of a reward-paired cue^20^.

As with incubation effects and DARPP32, few previous findings exist to compare the EE effects we observed. Gomez *et al*. reported that rats with 30 days of EE just after weaning had decreased Ratio (pThr34 DARPP32:DARPP32) values in the prefrontal cortex and nucleus accumbens (no sub-regions indicated)^21^. Lee *et al*. evaluated DARPP32 in several general brain systems after 2 months of chronic EE in mice; EE increased DARPP32 in the “frontal cortex” and “basal ganglia”^22^. In contrast, approximately 30 days of EE post weaning had no effect on measures of pThr34 DARPP32 in the whole nucleus accumbens^23^. In addition, there was no effect of chronic EE in mice on total DARPP32 in striatum or antero-medial cortex^24^. There are species, age, and contextual (including reinforcer availability) differences between these previous studies and our own. These differences could explain the variability across studies in observing effects of EE on cortical and basal ganglia DARPP32.

There were only two effects specific to whether subjects were tested or not. Both pThr34 DARPP32 and the Ratio value were increased in the prelimbic area of the mPFC in subjects that were not tested (Fig. 4). The functional significance of this difference is difficult to hypothesize, as the prelimbic cortex is associated with many cognitive and behavioral functions^25^. As the effect of Testing was to increase phosphorylation of DARPP32, it is conceivable that this reflected greater dopamine D1 receptor stimulation in this region due to greater dopamine overflow. Increased dopamine receptor activation could relate to greater attentional demand^26^ due to placement into the sucrose cue-rich Testing environment.

Regarding dopamine receptors, the present results provide only limited insight into the role of D1 receptors in the effects of EE on seeking behavior. Although our previous behavioral pharmacology findings indicate a role for D1 receptors in sucrose seeking, including incubation of sucrose seeking^6^ and the reduction in sucrose seeking following EE^7^, verification of a D1 receptor mechanism mediating the DARPP32 results in the present study will require further research. Furthermore, there are many potential mechanisms beyond D1 receptors to account for the effects we observed and this needs to be considered, especially as we noted differential effects of incubation and/or EE on DARPP32 across brain regions. For example there is a large family of mediators of DARPP32 phosphorylation sites (Thr34, Thr75, Ser97, Ser130) including dopamine D2^27^, NMDA glutamate^28,29^, and acetylcholine receptors^30^. We must emphasize that a lack of effect of testing, abstinence, and/or EE on phosphorylation of DARPP32 at Thr34 in the present study does not rule out a role for dopamine and/or DARPP32 in a particular brain region. For example, a nigrostriatal 6-OHDA lesion does not alter phosphorylation of DARPP32 at Thr34^31,32^, but it increases phosphorylation at Thr75^32^. There are also interactions between D1 and other receptor proteins. D1 receptors affect GABA neurotransmission^33^ and D1 agonism increases NMDA receptor trafficking^34^. DARPP32 itself mediates NMDA trafficking, and facilitates non-synaptic communication between neurons^35^. Connecting DARPP32 back to EE, it was recently reported that 24 h EE resulted in changes in spine morphology in the nucleus accumbens. The effect is mediated by DARPP32 and its interaction with adducin, a protein that regulates synaptic stability by capping actin filaments^36^. This last finding is just part of an expanding literature of effects of EE the structure and function of neurons and their connectivity. For example, EE has been found to have widespread effects on neuroplasticity markers c-fos^8,37,38^ and delta fos B^39^, and alters the intensity of perineuronal net staining in cortical regions^40^.

Especially relevant to the EE-mediated effects on DARPP32 in the nucleus accumbens core, studies could delineate effects specific to direct or indirect projection pathways^41^ or ensembles affected by incubation and/or EE^42^. In addition, functional roles of the protein changes we observed should be conducted, although at this time there is no ligand specific for the DARPP32 protein other than antibodies. Instead, site-directed manipulations targeting dopamine or glutamate receptors in the nucleus accumbens core, or even other regions where we observed changes in pThr32 DARPP32 due to EE, could reveal how changes upstream from DARPP32 affect sucrose seeking. In addition, and as noted above, other phosphorylation sites of DARPP32 (e.g. Thr75) and molecules that alter phosphorylation of DARPP32 (e.g. protein kinase A and cyclin-dependent-like kinase 5) should be examined. Such studies will help elucidate how EE, a non-pharmacological environmental “treatment”, has such a robust effect at reducing relapse behavior.

In conclusion, the effects we measured suggest a complex interplay in signaling across brain regions that relates to, and perhaps mediates, motivation to seek sucrose. Further study is required, including examining the generalizability of these results to other reinforcers including drugs of abuse^5^ and identifying a potential role for glutamate and, possibly dopamine/glutamate interactions in mediating the effects of abstinence and EE on DARPP32 and pThr34 DARPP32. The results of these studies may lead to a better understanding of the molecular biology of relapse behavior and thus facilitate development of novel relapse treatment approaches.

## Methods

### Subjects

Subjects were 142 male Long-Evans rats bred in the Western Washington University vivarium, approximately 3 months old at start of study. Group sizes were n = 12–14, a range to allow for attrition and provide statistical power^10^. Rats were housed individually in Micro-Isolator chambers (20 × 32 × 20 cm; Lab Products, Inc., Seaford, DE) under a 12-h reverse day/night cycle with lights off at 0700 h. Purina Mills Inc. Mazuri Rodent Pellets (Saint Louis, MO) and water were available *ad libitum* throughout the study, except for water deprivation 17 h prior to the first Training session. Operant procedures occurred between 0900–1100 h. Weights were recorded every Monday, Wednesday, and Friday. All procedures followed NIH guidelines^43^ and were approved by the Western Washington University IACUC.

### Operant conditioning apparatus

Operant conditioning chambers (30 × 20 × 24 cm; Med Associates, St. Albans, VT) were equipped with one retractable lever to the left side of the reward receptacle where sucrose solution was dispensed. An inactive lever was located on the opposite wall. Chambers were equipped with four infrared photobeam emitters and detectors, a red house light, and a sound generator (2 kHz, 15 dB over ambient noise). Chambers were sound-attenuated and equipped with fans.

### Operant conditioning procedures

#### Training

Sessions began with illumination of the house light and insertion of the retractable lever. Rats underwent 10 daily 2-h sessions wherein they pressed the retractable lever for a 0.2 mL delivery of 10% sucrose reinforced under a fixed-ratio 1 schedule with a 40 s time-out. An active lever press was accompanied with a 5 s combined presentation of the white stimulus light and the tone along with sucrose. For this 5 s and the following 35 s, active lever presses were recorded but not reinforced.

#### Abstinence

Rats were randomly assigned to a condition consisting of a cross between duration of abstinence and type of housing condition. The abstinence period was either from the end of the tenth Training session to a Testing session the next morning (22 h; “Day 1”) or to a Testing session 30 days later (“Day 30”).

#### Environmental enrichment

EE consisted of a mixture of housing and social enrichment^10^. Three rats were housed together in EE.

EE was either acute (EEAcute) or chronic (EEChronic). EEAcute groups were placed into EE for 22 h prior to Testing. EE began either after training on the tenth day of Training, or at the same time of day on the 29^th^ day of abstinence (Day 1 and Day 30 EEAcute). EEChronic was exposure to EE from the end of the tenth day Training session until Testing on Day 30. All Control (CON) rats remained single-housed.

#### Testing

Following Training, rats were pseudo-randomly assigned to either a NO TEST or TEST condition. TEST conditions were identical to Training, except the syringe containing sucrose solution was absent and the session was 1 h. Rats in the NO TEST condition were not returned to the Testing environment prior to brain extraction.

### Western blot

Rats were rapidly decapitated immediately following testing (TEST) or at the same time of day (NO TEST). Brains were extracted within 80 sec and immediately snap frozen in 2-methyl butane previously chilled with dry ice. This latency from decapitation to freezing is similar to previous studies relating DARPP32 phosphorylation to behavior in rats and mice^14,44^. This latency is longer than in studies where investigators anesthetized mice and froze their heads immediately before brain extraction^45^. Thus, on one hand our results reflect DARPP32 and pThr34-DARPP32 during the rapidly declining phase of neural activity and glutamate transmission 80 seconds following behavior, but this is not as long as the time between behavior and tissue preservation if using anesthesia (many minutes)^45^. In addition, our method avoided use of anesthesia that rapidly alters glutamatergic input to neurons and thereby likely alters DARPP-32 phosphorylation state from that found in the behaving rat. One mm frozen coronal sections were taken at the following anterior/posterior coordinates relative to bregma^46^: +3.2, +1.6, and −5.6 mm. Bilateral punches of prelimbic, infralimbic, and anterior cingulate areas of the medial prefrontal cortex, orbitofrontal cortex, dorsomedial and dorsolateral striatum, core and shell of the nucleus accumbens, and ventral tegmental area (VTA) were taken using stainless steel needles (13G for accumbens shell, 16G for VTA, and 15G for all other regions). Punches were stored at −80 °C. Supplement 1 indicates approximate locations of punches.

Punches were later prepared for Western blot following Theberge and colleagues^47^. Punches were manually homogenized using pestles in microtubes (Bel-Art, Wayne, NJ) containing RIPA lysis buffer (Cell Signaling, Danvers, MA) along with phosphatase (PhosSTOP, Roche, Indianapolis, IN) and protease (Complete Ultra, EDTA-free, Roche) inhibitors followed by 5, 2 s 50% amplitude pulses of sonication. After centrifugation (14,000 rpm) at room temperature for 10 min, protein concentration of supernatant was measured (Pierce BCA, Thermo Fisher Scientific, Rockford, IL). Calculation for a 25 µL, 1 µg/µL total protein sample containing 5 µL sample buffer and 2 µL DTT was then done. Sample plus lysis buffer was heated at 70 °C for 1 h. NuPAGE LDS sample buffer (Invitrogen, Carlsbad, CA) was added and samples were stored at −80 °C.

After thawing, samples were diluted to 1 µg/µL with DTT and heated at 90 °C for 5 min. At room temperature, 25 µL samples were loaded into wells in mini Bis-Tris precast 10% polyacrylamide gels (NuPAGE) and electrophoresed at 150 V for 1 h 45 min. The running buffer was NuPAGE MOPS SDS with 500 µL NuPAGE antioxidant added to the inner chamber. Proteins were then transferred to PVDF Immobilon-FL membranes (Millipore, Billerica, MA) for 1 h at 30 V in NuPAGE transfer buffer and 1 mL/L NuPAGE antioxidant. Brain regions were electrophoresed on separate runs of gels, with experimental conditions represented randomly across gels.

Membranes were probed for DARPP32 and pThr34 DARPP32 following the Li-Cor Western Blot protocol (Li-Cor, Lincoln, NE). Membranes were incubated in blocking buffer (Li-Cor) for 1 hour at room temperature and then in primary antibodies overnight at 4 °C. Primary antibodies were 1:100 mouse monocolonal DARPP32 (H-3, sc-271111, lot B1815, Santa Cruz Biotechnology, Dallas, TX) and 1:100 goat polyclonal Thr34 DARPP32 (sc-21601, lot J2915, Santa Cruz Biotechnology). Secondary antibody incubation followed at room temperature for 45 min. Secondary antibodies were 1:10,000 IRDye 680 RD donkey anti-goat (Li-Cor) and 1:10,000 IRDye 800 CW donkey anti-mouse (Li-Cor). Fluorescence intensity was detected using a Li-Cor Odyssey Fc infrared imaging system running Image Studio 5.2 software (Li-Cor). After imaging, membranes were stripped using NewBlot IR stripping buffer (Li-Cor) and stained using REVERT Total Protein Stain (Li-Cor). Membranes were imaged for total protein in each lane. Intensity values for total proteins were then normalized across each membrane. DARPP32 and pThr34 DARPP32 intensity values were corrected using the normalization value for their respective lanes. This provided values relative to total protein in a sample^48^. Brain regions were assayed separately, using fresh aliquots of primary antibodies for each region. A representative membrane, imaged for DARPP32 and pThr34 DARPP32 and then for total protein, is provided in Supplement 1. Representative blots from each of the 9 brain regions examined are also provided in Supplement 1. Representative blots are provided for each condition on data Figures. A representative blot is the median value for a condition. If a condition had an even number of data points, the value to the left of the middle of the distribution was used. If the chosen value had a normalization value of less than 0.7, the value nearest to it with the largest normalization value was used. Normalization values for all representative blots averaged 0.9.

### Statistical analyses

For Day 1 of abstinence there were two Housing conditions (CON, EEAcute). For Day 30 of abstinence there were three Housing conditions (CON, EEAcute, EEChronic). To account for the missing cell (no Day 1 “chronic” EE), the Type IV SS model was used for analysis of variance (ANOVA)^49^. Training data were analyzed to ensure groups did not differ prior to treatment assignment. Measures of responding (active lever, sucrose infusions, inactive lever, photobeam breaks) over the final 5 days of Training were analyzed separately using repeated measures ANOVA. For rats that were tested for cue reactivity, these same Testing measures were analyzed separately using ANOVA. For each brain region, separate ANOVAs were calculated for DARPP32, pThr34 DARPP32, and the ratio of pThr34 DARPP32 to DARPP32 (“Ratio” in Table and Figures). Regions were analyzed separately as they were processed separately. For this reason, between-region comparisons were not made using inferential statistics. Pre-training body weights were analyzed to determine if groups differed prior to treatment assignment. Weights were later compared between Day 30 Testing groups to determine if chronic EE affected body weight.

A main effect or interaction of TEST prompted separate ANOVAs for TEST and NO TEST subjects. Statistically significant main effects for EE in ANOVA were followed by Šidák-adjusted post-hoc tests. Following a significant DAY X HOUSING interaction, one-tailed t-tests were done: tests were between Day 1 CON and Day 30 CON, Day 1 CON and Day 1 EEAcute, Day 30 CON and Day 30 EEAcute, and Day 30 CON and Day 30 EEChronic, resulting in 4 comparisons. Family-wise error for these tests was reduced by using a Šidák correction, resulting in a criterion for statistical significance of *p* < 0.0127. For all other statistical comparisons, *p* < 0.05 was used. For TEST subjects, Pearson r correlations were calculated for each brain region separately, assessing the degree of association between intensity measures and active lever responding during the cue-reactivity test.

IBM SPSS Statistics 24 was used for all statistical calculations except t-tests were calculated in Excel 2016. Figures were created in Excel 2016. Western blot results are expressed as arbitrary units.

### Data Availability

The datasets generated during and/or analyzed during the current study are available from the corresponding author on reasonable request.

## Electronic supplementary material

Supplementary Information
