# Supplementary material for: Sucrose Abstinence and Environmental Enrichment Effects on Mesocorticolimbic DARPP32 in Rats
Source: Sci Rep. 2018 Sep 4;8:13174. doi: 10.1038/s41598-018-29625-x (PMC6123458; doi:10.1038/s41598-018-29625-x)

Supplement 1

Supplementary Information for:
Sucrose Abstinence and Environmental Enrichment Effects on Mesocorticolimbic DARPP32 in Rats

Author list and affiliations:
Jeffrey W. Grimm*, Edwin Glueck, Darren Ginder, Jeff Hyde, Katherine North, and Kyle Jiganti
Department of Psychology and Program in Behavioral Neuroscience
Western Washington University

Corresponding author*:
Department of Psychology and Program in Behavioral Neuroscience
Western Washington University
516 High Street
Bellingham, WA 98225-9172
phone: 360-650-3168
fax: 360-650-7305
jeff.grimm@wwu.edu
ORCID iD 0000-0001-5316-5659

Supplement 1 Figure 1. Micropunches. Approximate regions[46] where bilateral micropunches were collected from 1 mm thick slices. Millimeters from bregma are indicated for each slice. Abbreviations: prelimbic cortex (PL), orbitofrontal cortex (OF), infralimbic cortex (IL), anterior cingulate cortex (AC), dorsomedial striatum (DM), dorsolateral striatum (DL), nucleus accumbens core (C), nucleus accumbens shell (S), and ventral tegmental area (VTA).

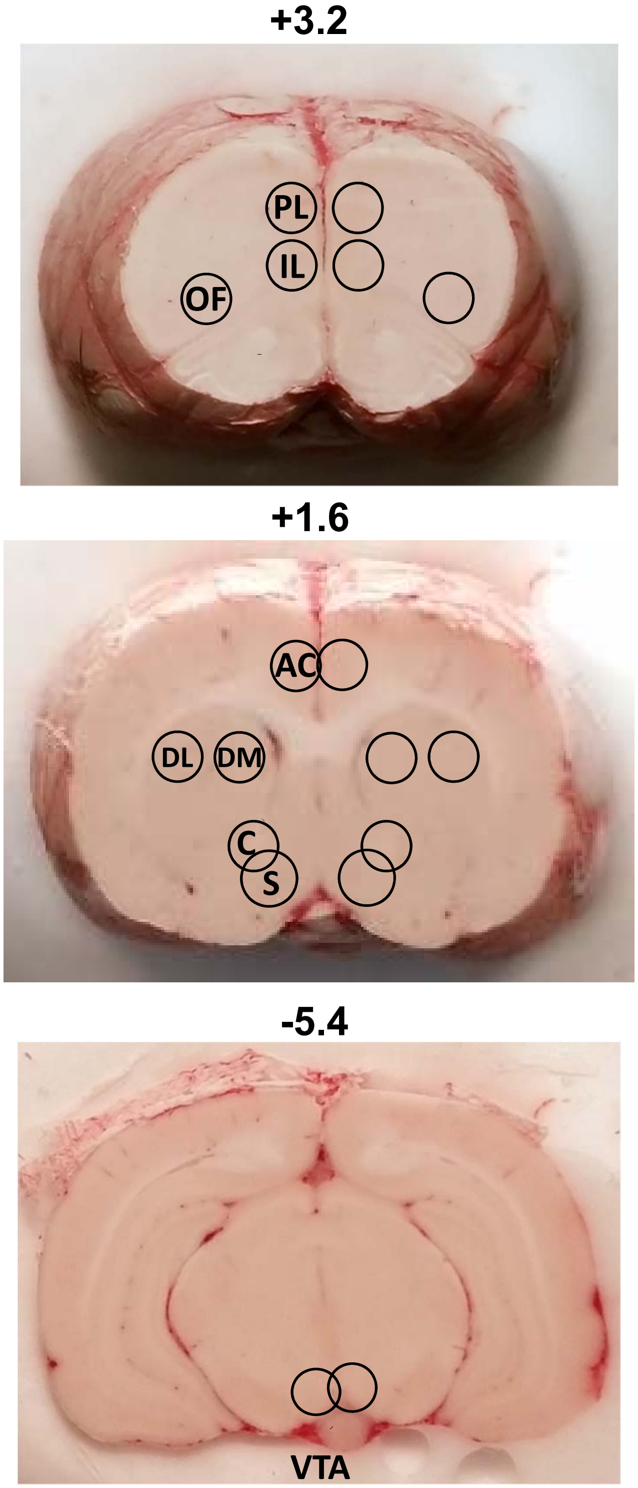

Supplement 1 Figure 2. Representative membrane. Top: membrane was incubated with antibodies for DARPP32 (lower, green band) and pThr34 DARPP32 (upper, red band). SeeBlue (Invitrogen) pre-stained protein standards are in lanes 5 and 10 from left. The MW 28 kDa marker is indicated. Bottom: the same membrane stained for total protein (see Method) to provide a loading/transfer control.

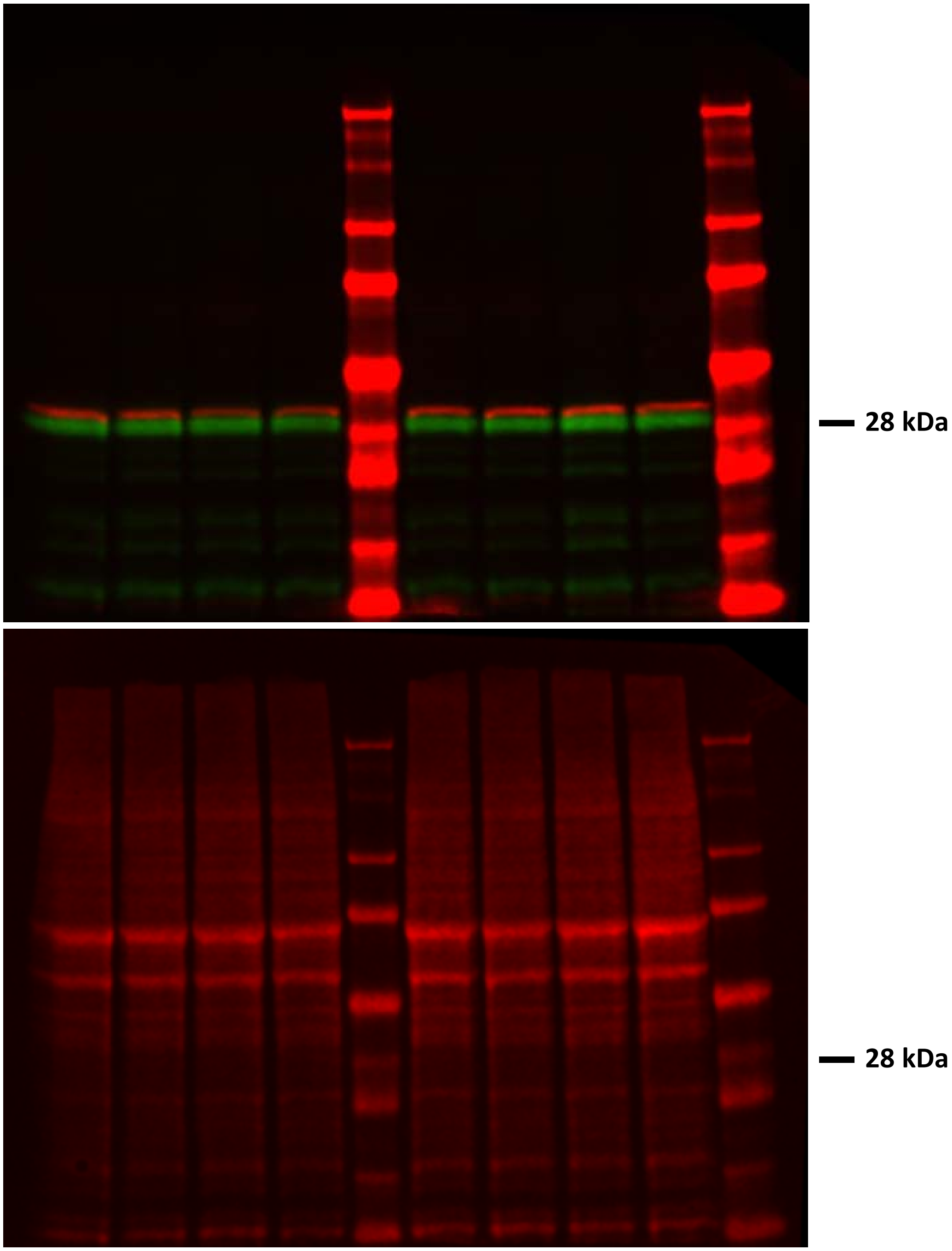

Supplement 1 Figure 3. Representative blots from the 9 brain regions examined. Blots are from different membranes. The approximate location of the 28 kDa molecular weight marker is indicated next to OF. Migrations were similar across all brain regions. Each blot represents the median intensity DARPP32 data point from the NO TEST CON Day 1 condition. The value to the left of median was used for groups with an even number of subjects. Abbreviations: prelimbic cortex (PL), infralimbic cortex (IL), orbitofrontal cortex (OF), anterior cingulate cortex (AC), dorsolateral striatum (DL), dorsomedial striatum (DM), nucleus accumbens core (C), nucleus accumbens shell (S), and ventral tegmental area (VTA).

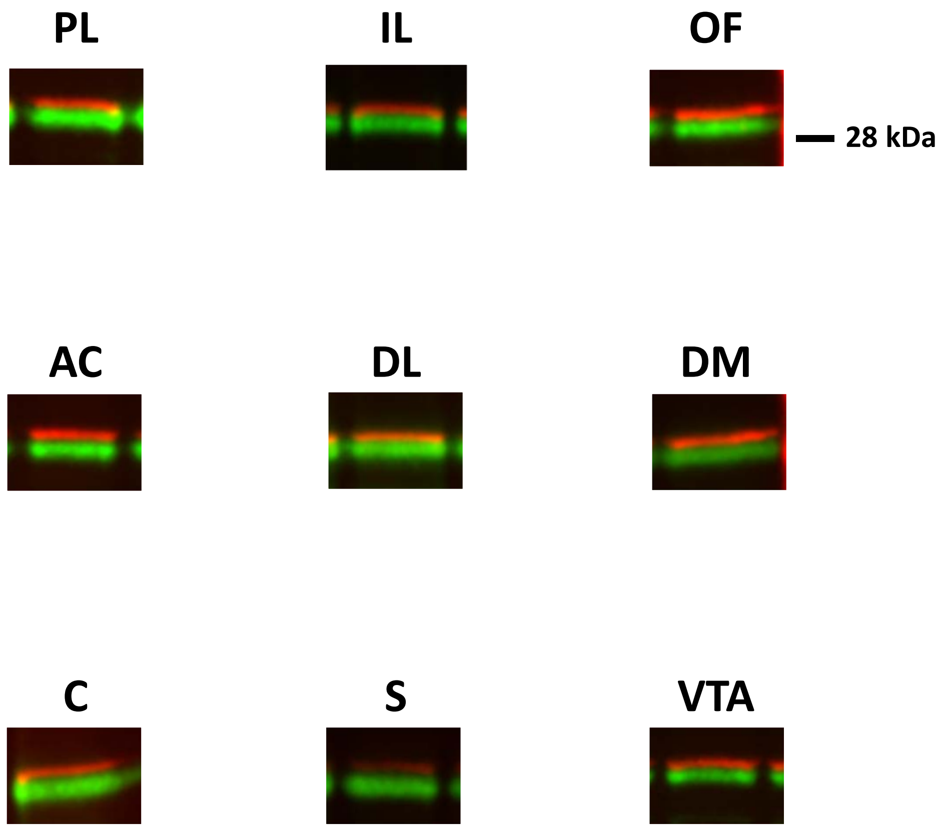

Supplement 1 Figure 4. Native (full) gel blots. In some instances either Total or phosphorylated protein is shown. In other instances, both are shown. This is to match with the presentation of sample blots in Supplement 1 Figure 3 and manuscript Figures. Molecular weight marker is visible only in images from the 700 nm channel. For 800 nm images, migration of the target protein (DARPP32) was verified by first viewing both channels simultaneously. The Figure to where the blot (outlined in red) is found is indicated above each full-length blot. The experimental condition is either indicated along the top of the blot, or on the blot if more than one condition is represented.

Supplement Fig. 3 (PL)

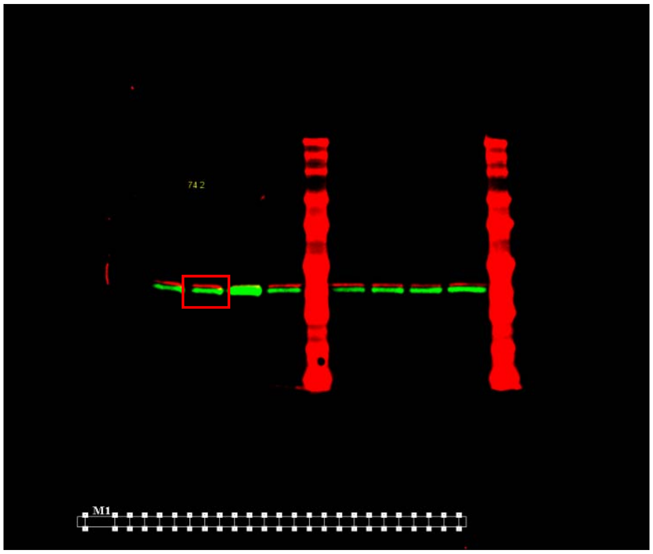

Supplement Fig. 3 (IL)

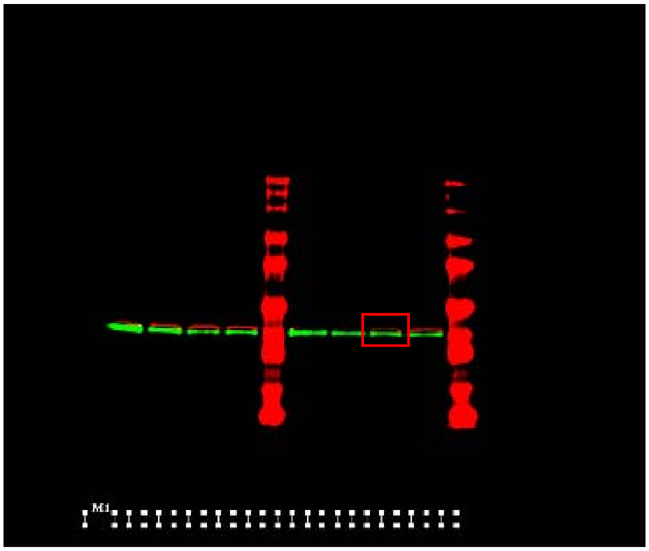

Supplement Fig. 3 (OF)

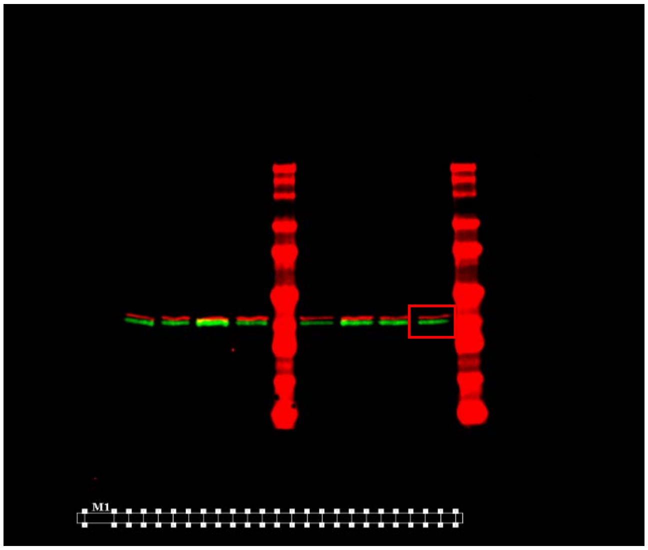

Supplement Fig. 3 (AC)

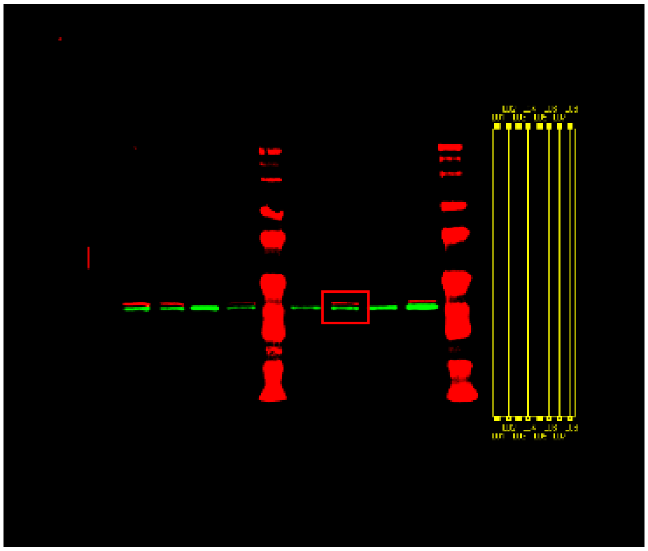

Supplement Fig. 3 (DL)

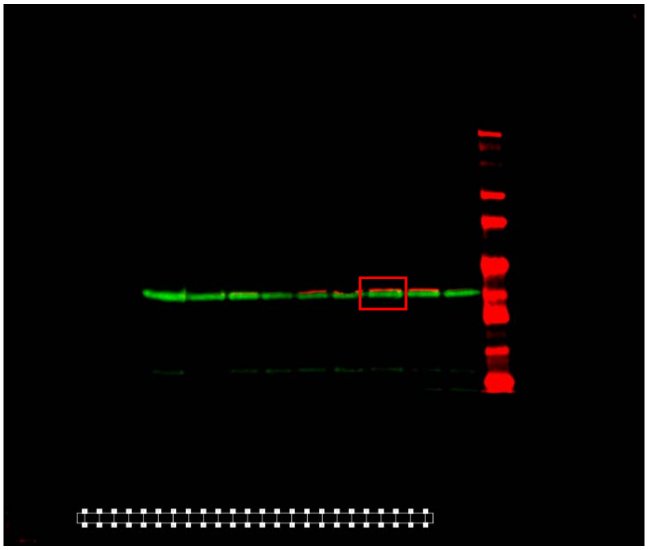

Supplement Fig. 3 (DM)

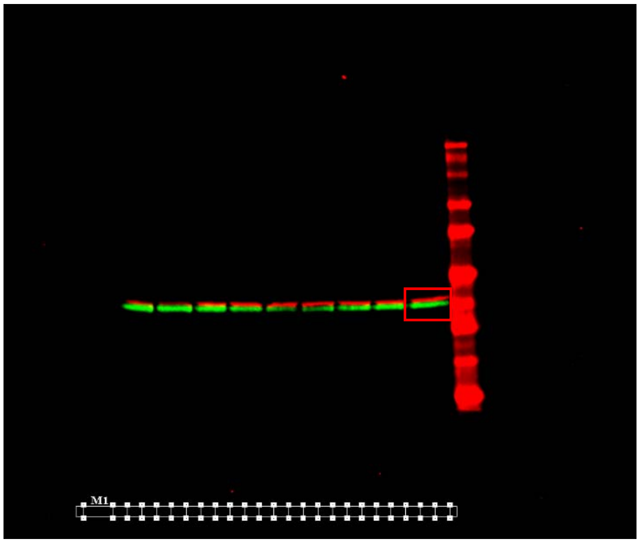

Supplement Fig. 3 (C)

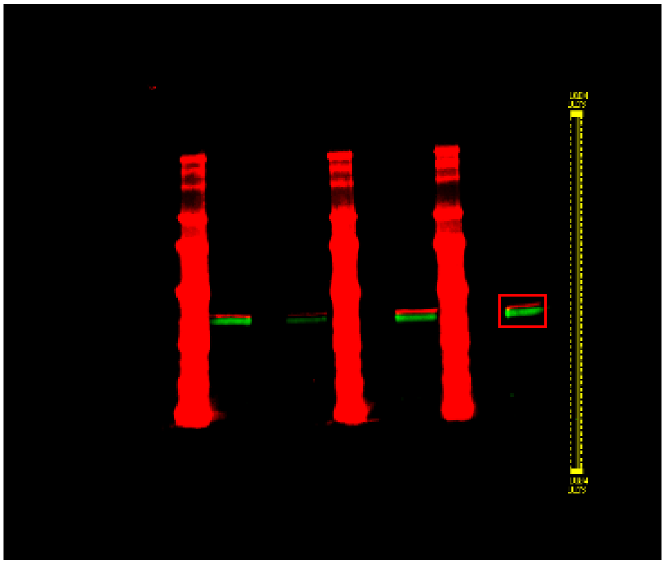

Supplement Fig. 3 (S)

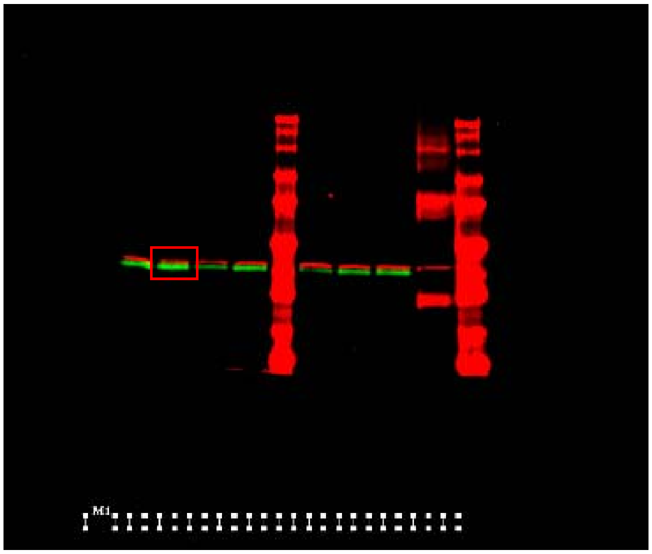

Supplement Fig. 3 (VTA)

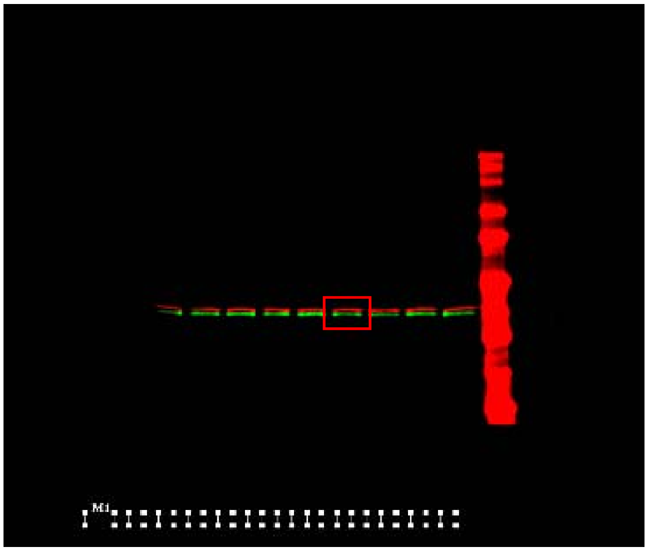

Fig. 4A NO TEST

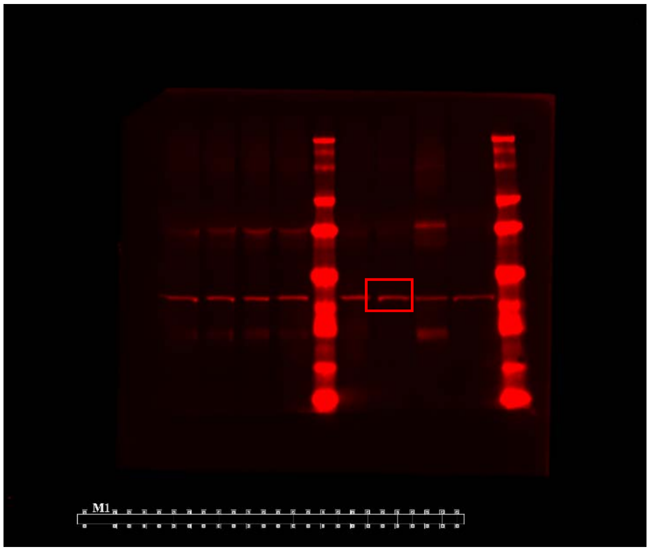

Fig. 4A TEST

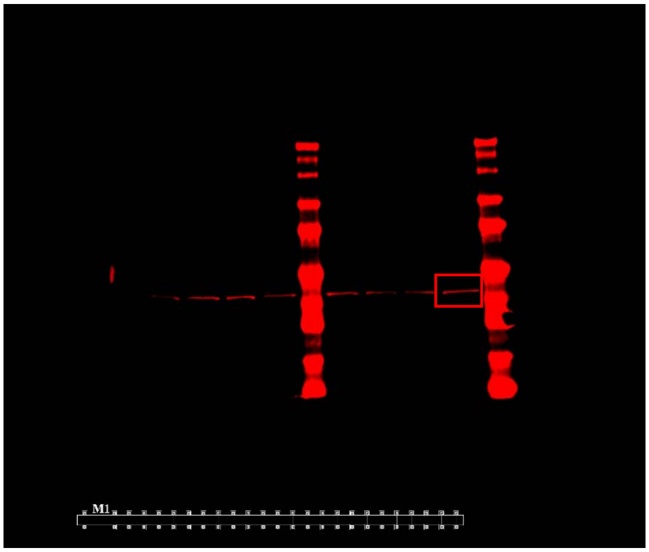

Fig. 4B NO TEST

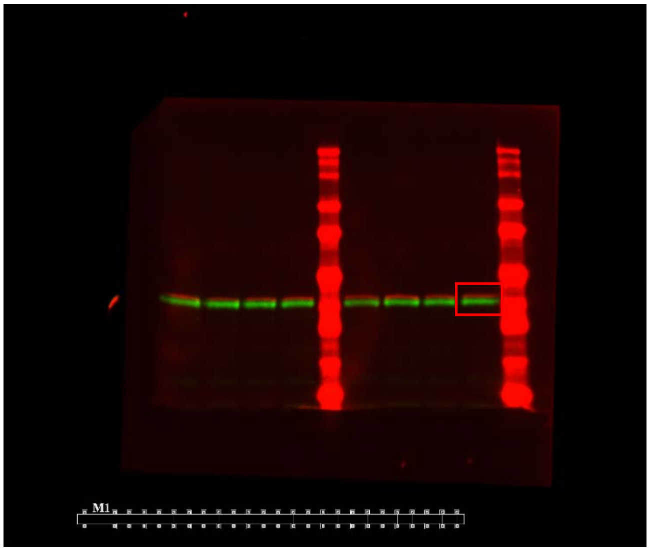

Fig. 4B TEST

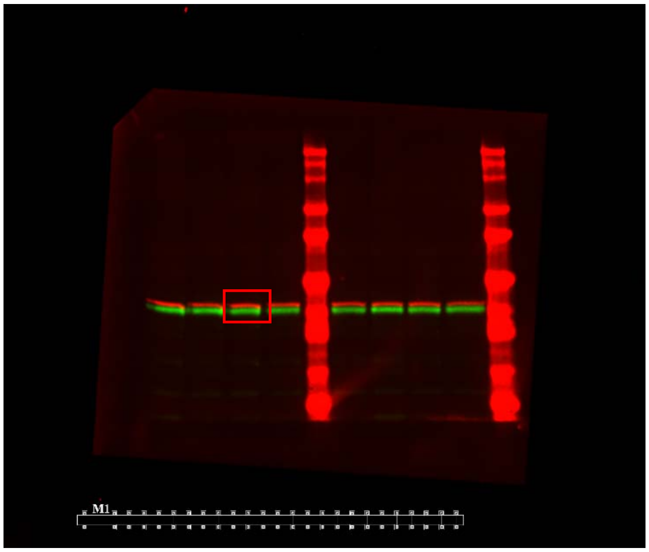

Fig. 5A Day 1 CON

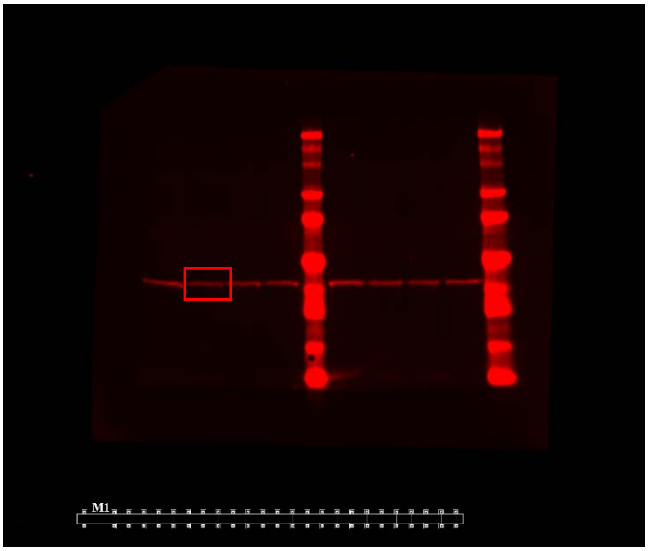

Fig. 5A Day 1 EEAcute

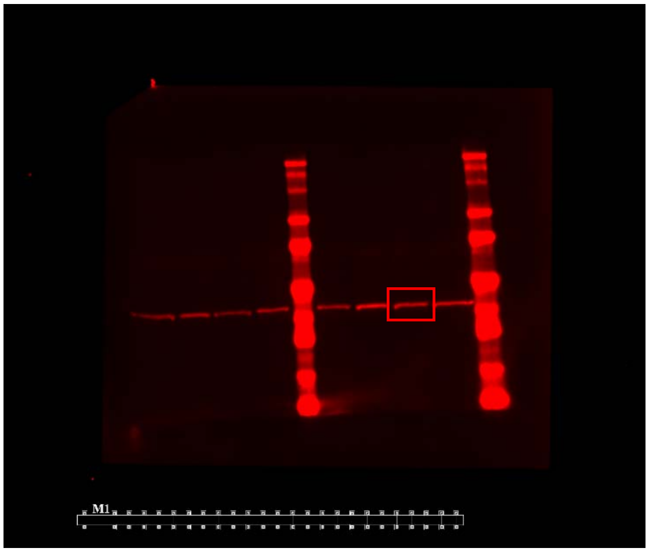

Fig. 5A Day 30 CON

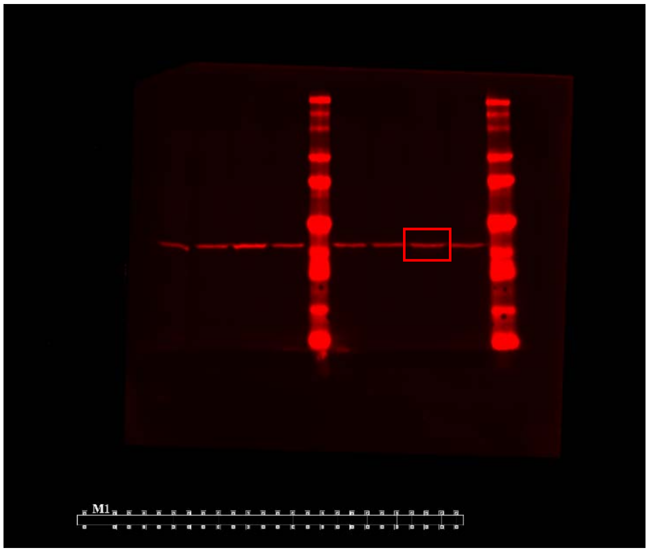

Fig. 5A Day 30 EEAcute

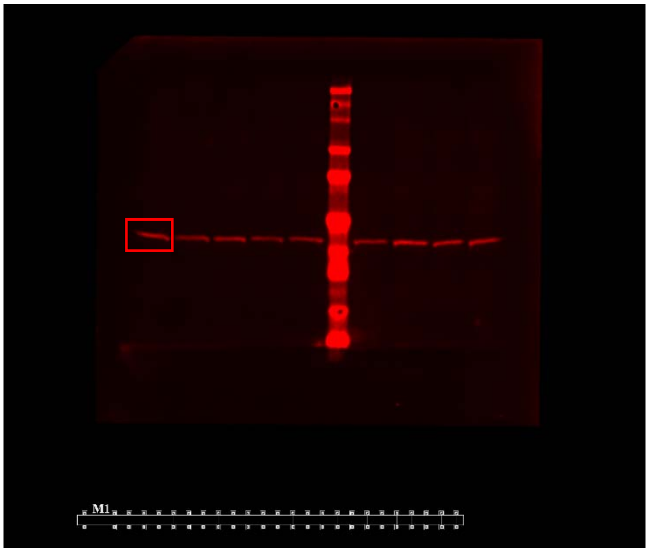

Fig. 5A Day 30 EEChronic

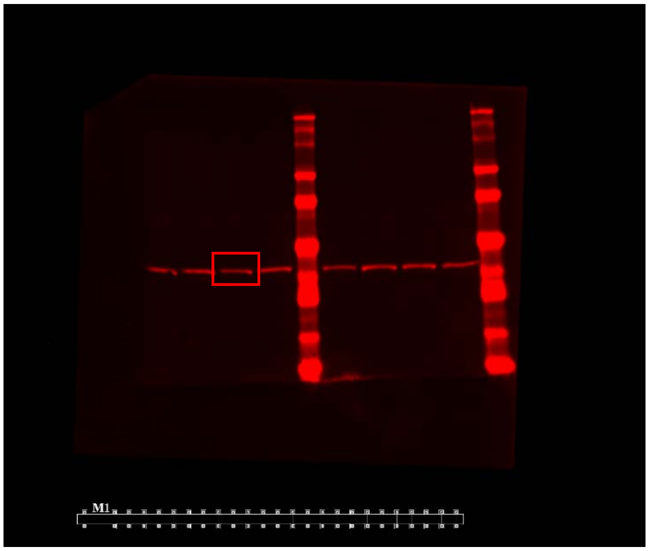

Fig. 5B Day 1

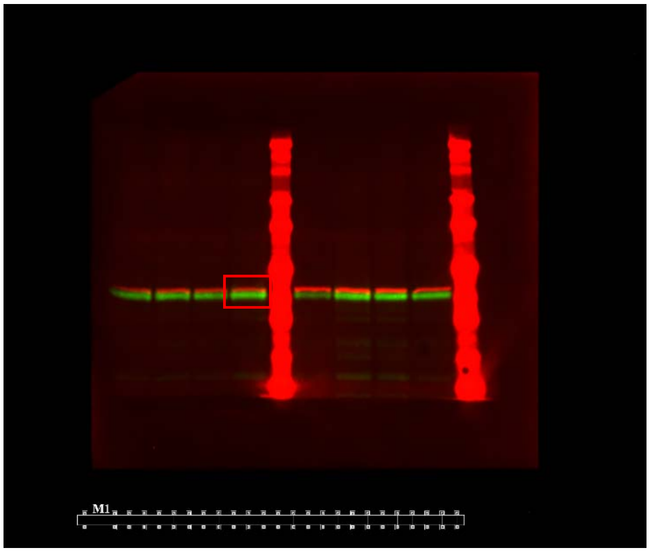

Fig. 5B Day 30

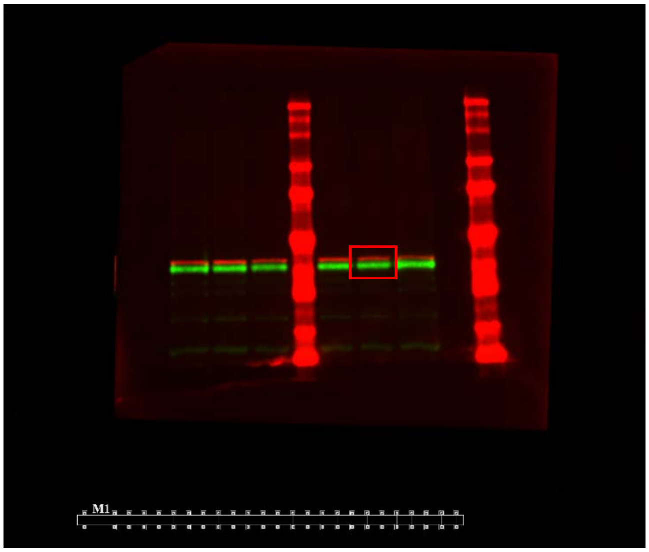

Fig. 6A Day 1 CON

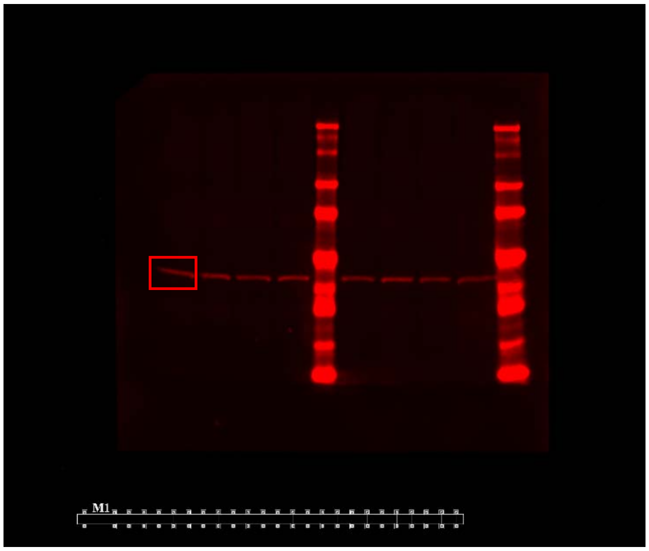

Fig. 6A Day 1 EEAcute

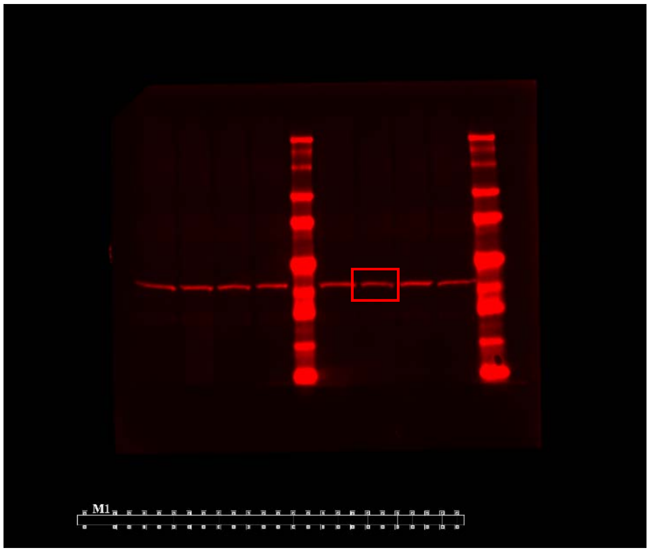

Fig. 6A Day 30 CON

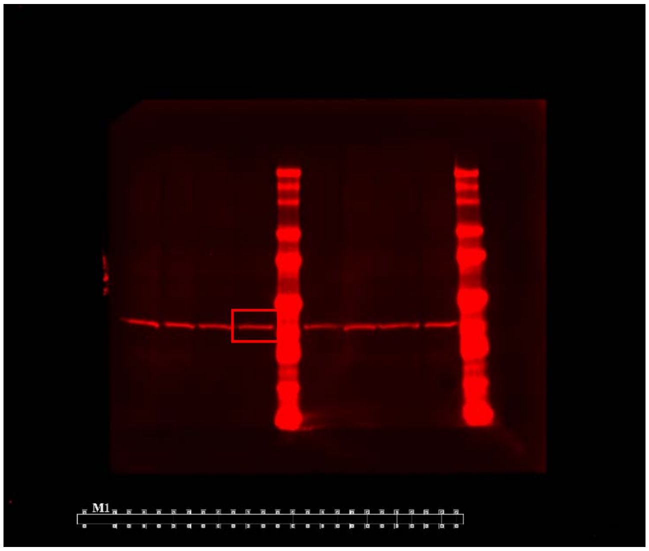

Fig. 6A Day 30 EEAcute

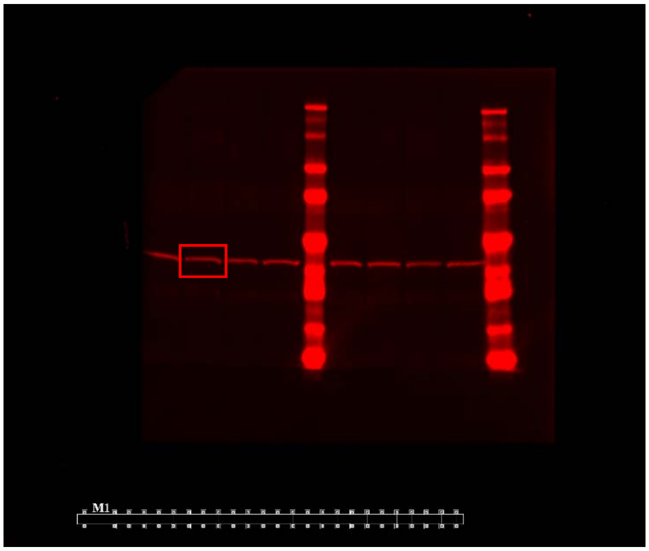

Fig. 6A Day 30 EEChronic

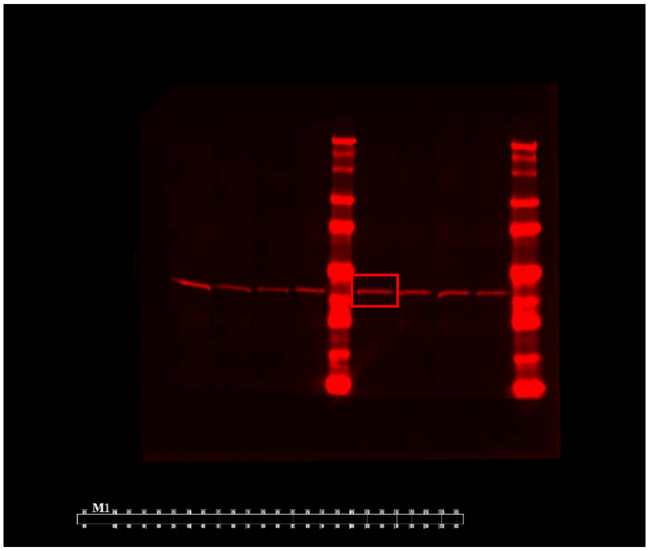

Fig. 6B Day 1 CON

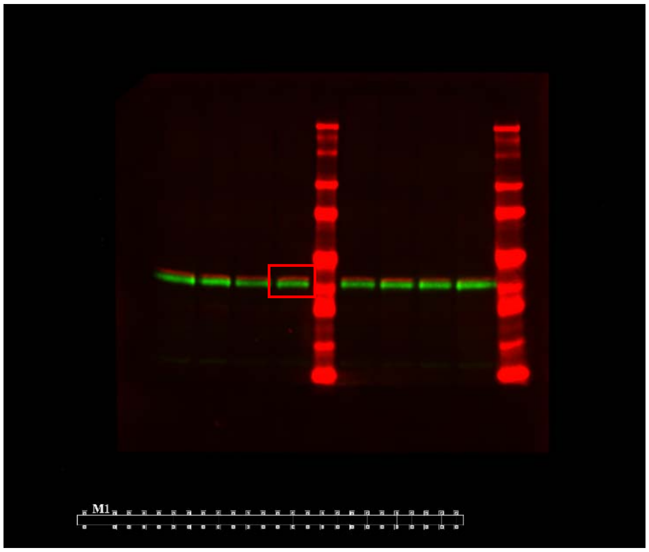

Fig. 6B Day 1 EEAcute

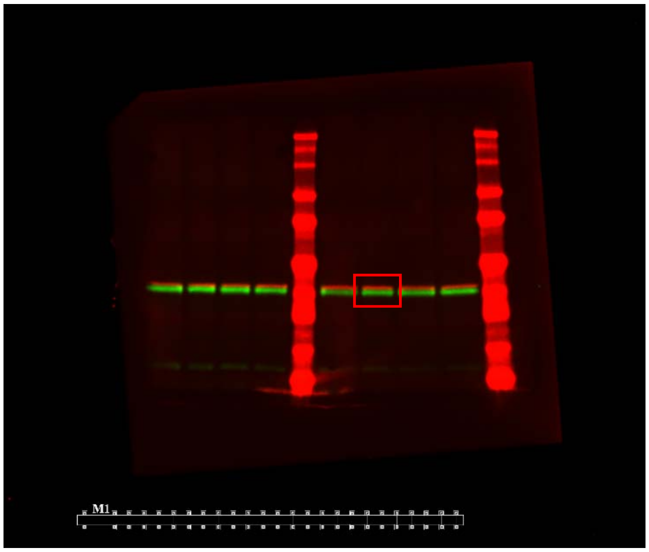

Fig. 6B

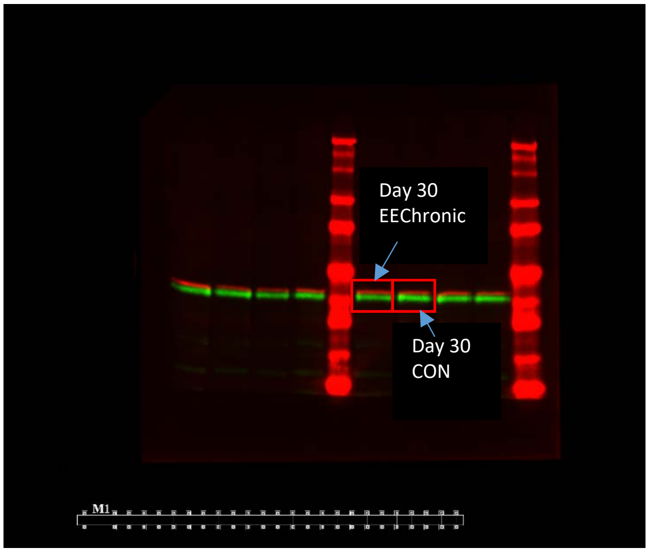

Fig. 6B Day 30 EEAcute

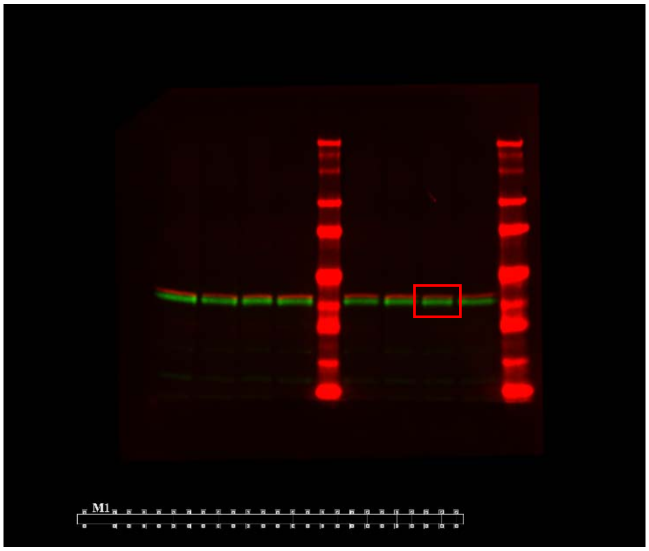

Fig. 7A Day 1 CON

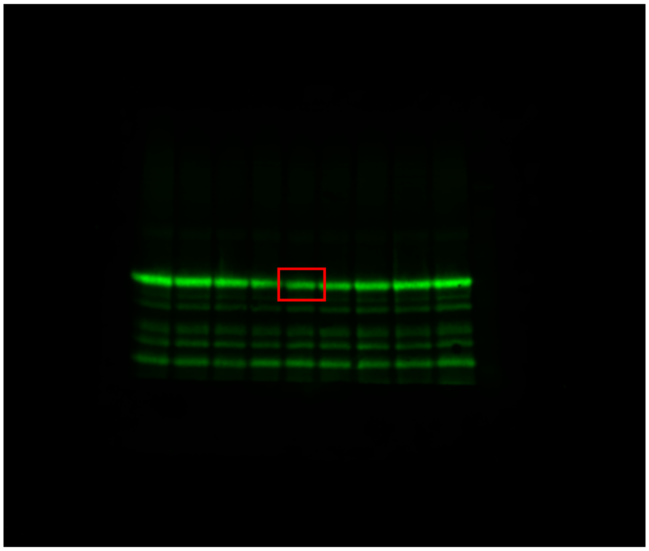

Fig. 7A

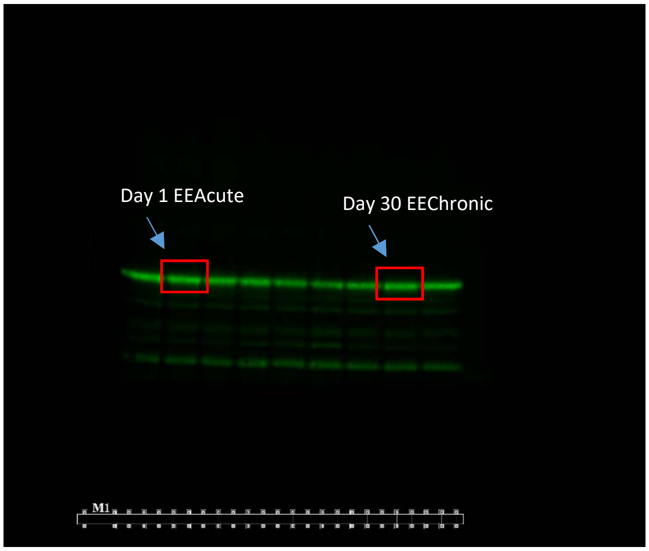

Fig. 7A Day 30 CON

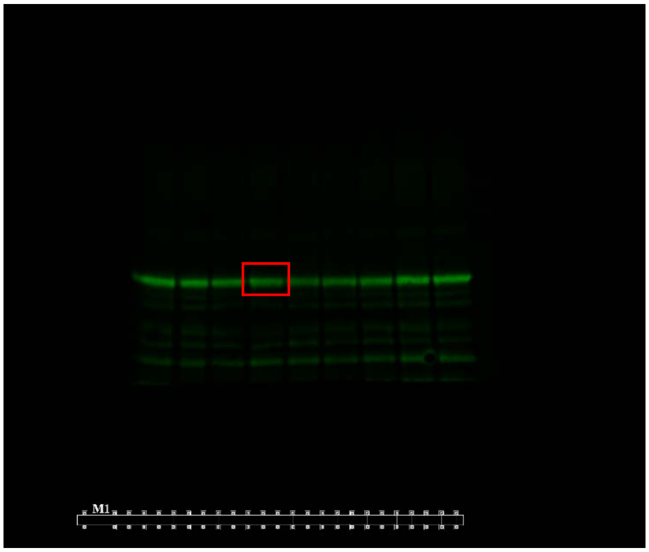

Fig. 7A Day 30 EEAcute

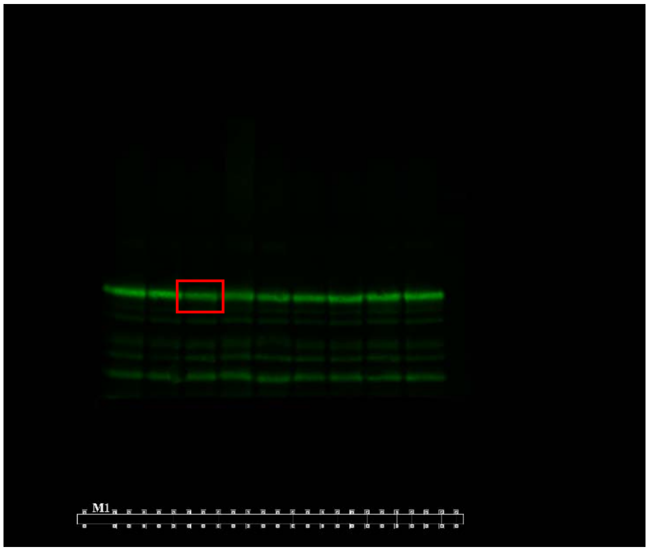

Fig. 7B Day 1 CON

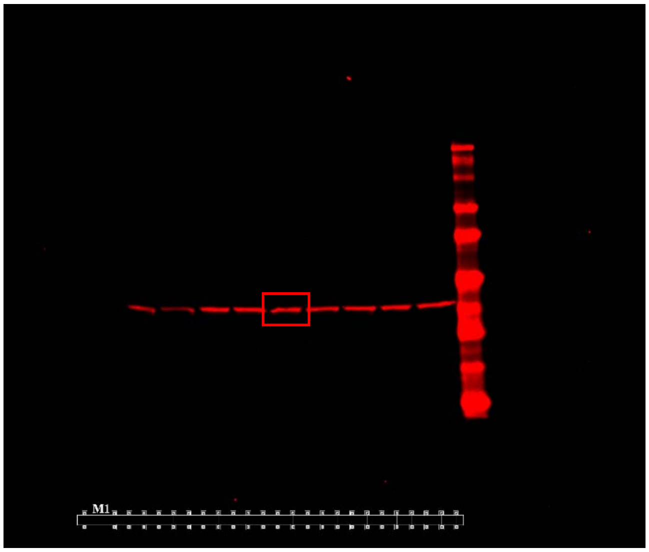

Fig. 7B Day 1 EEAcute

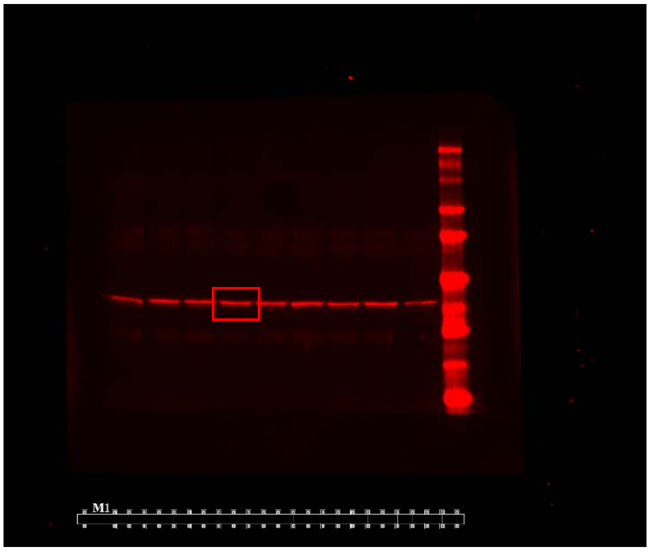

Fig. 7B Day 30 CON

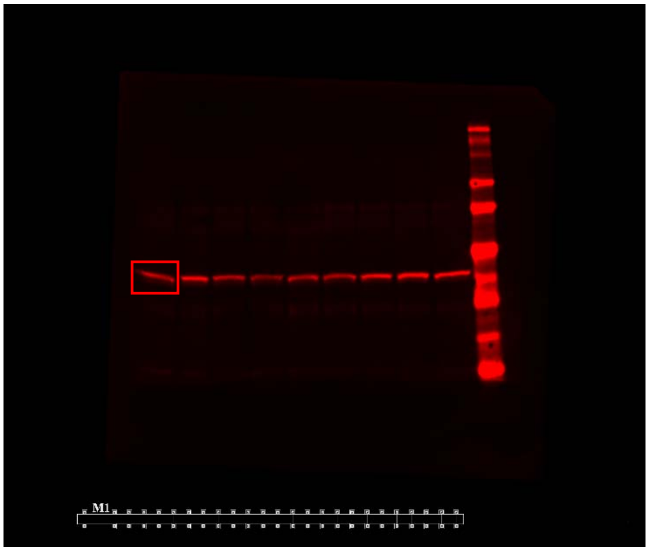

Fig. 7B Day 30 EEAcute

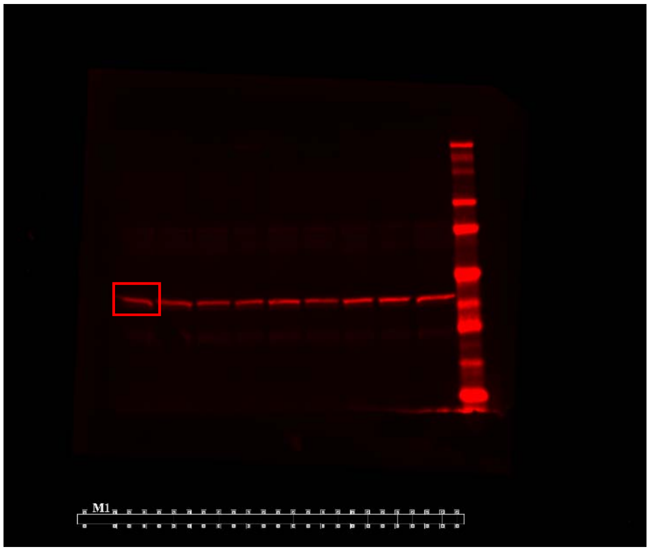

Fig. 7B Day 30 EEChronic

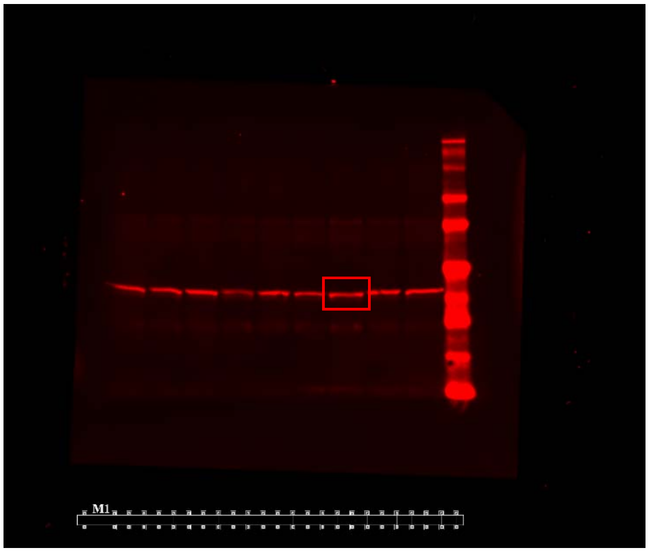

Fig. 8A CON

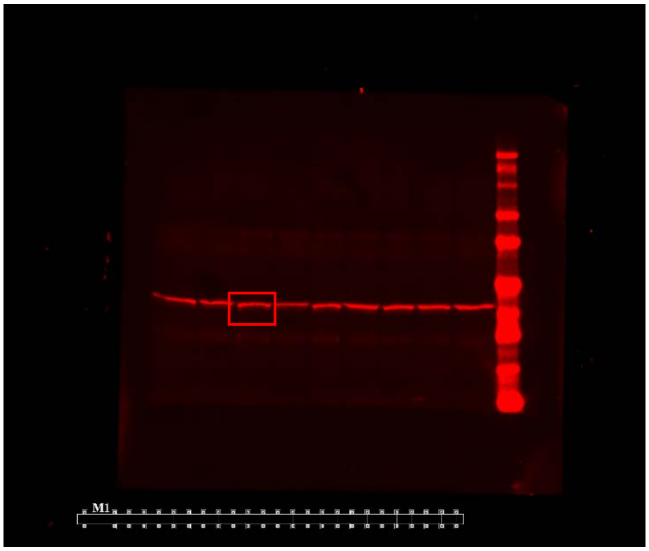

Fig. 8A EEAcute

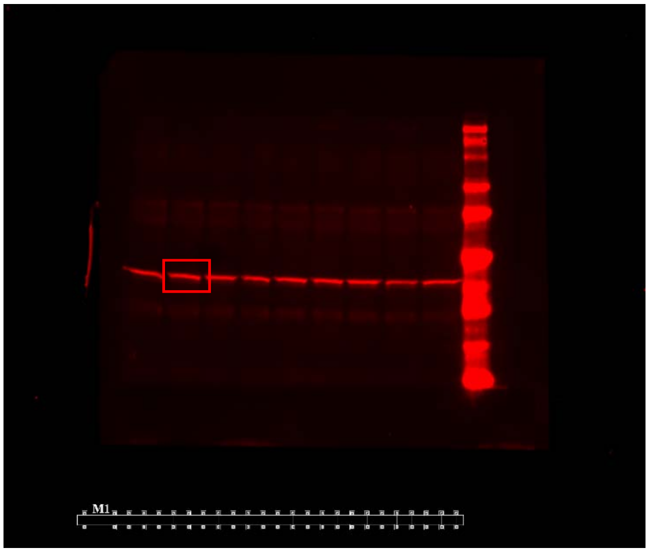

Fig. 8A EEChronic

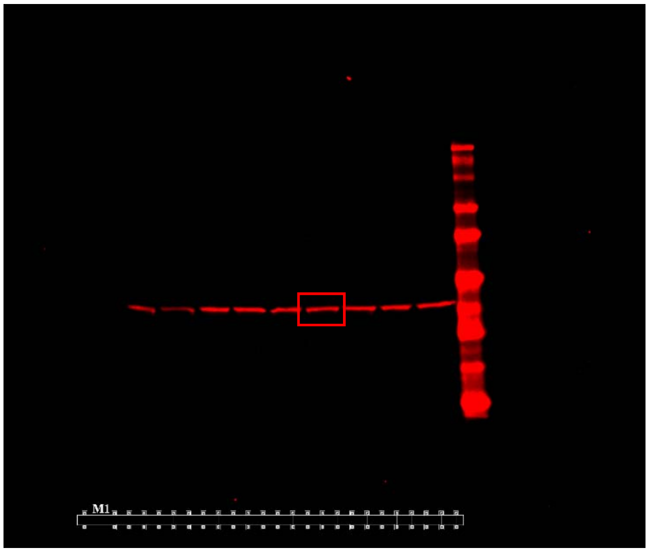

Fig. 8B Day 1

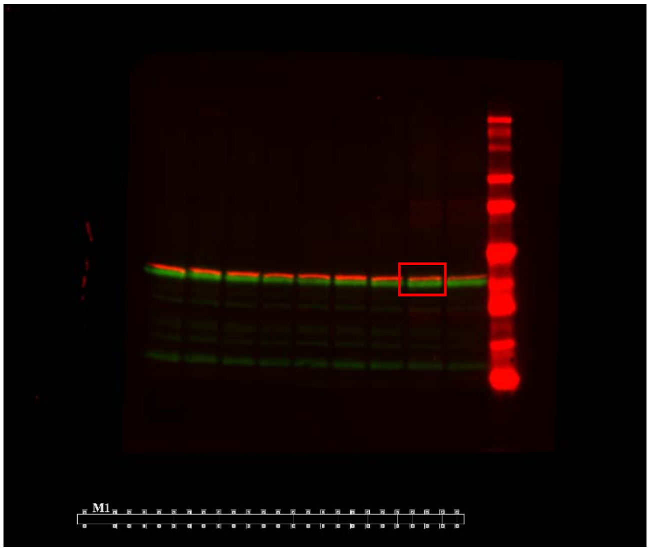

Fig. 8B Day 30

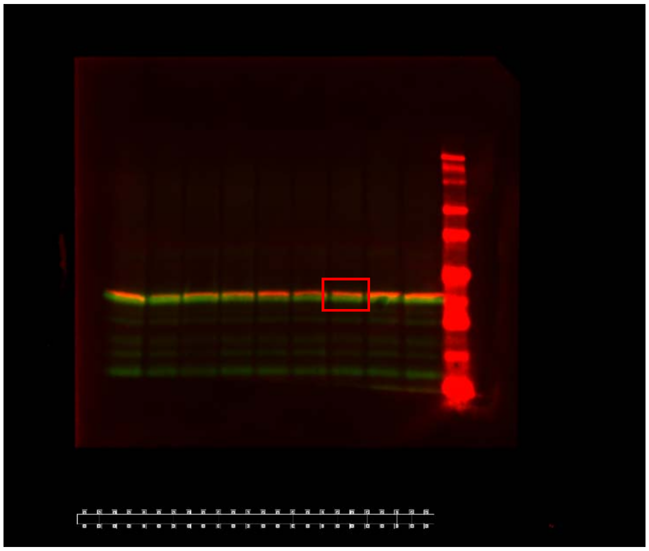

Fig. 9A CON

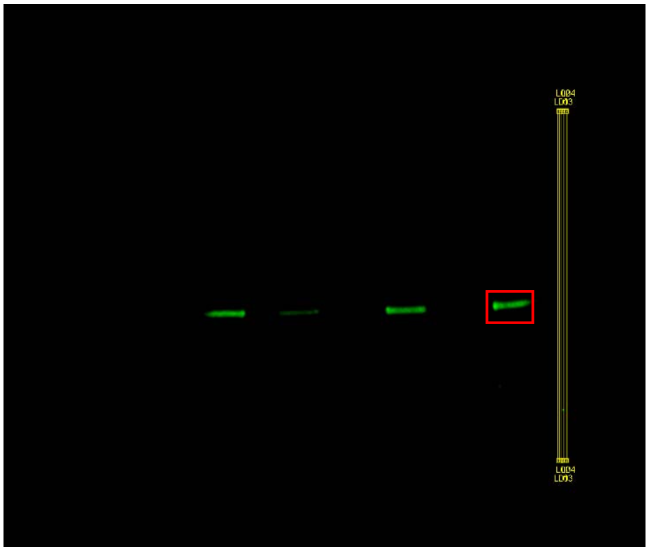

Fig. 9A EEAcute

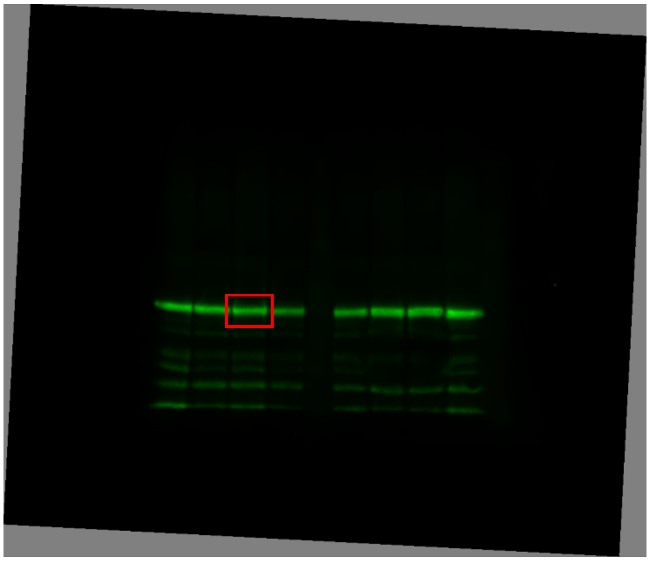

Fig. 9A EEChronic

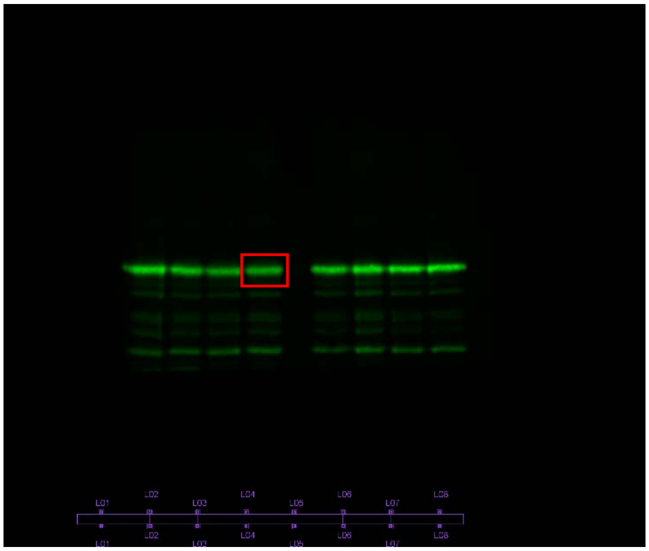

Fig. 9B CON

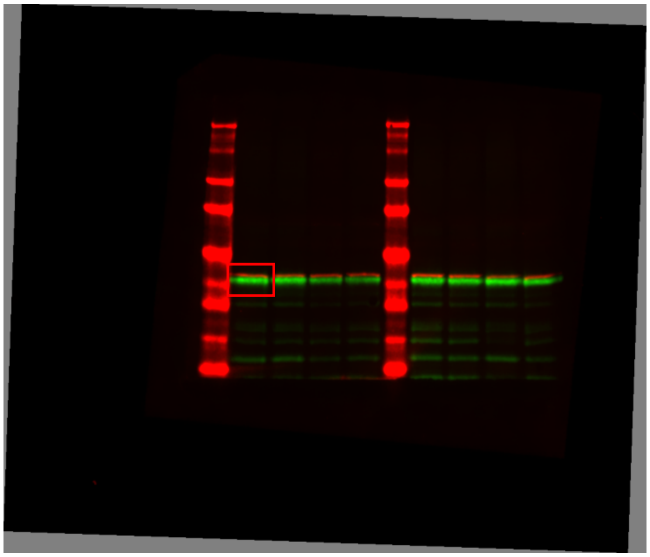

Fig. 9B EEAcute

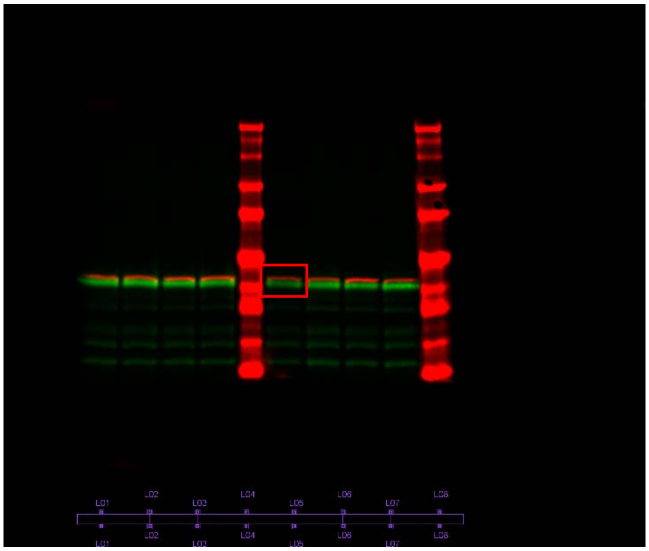

Fig. 9B EEChronic

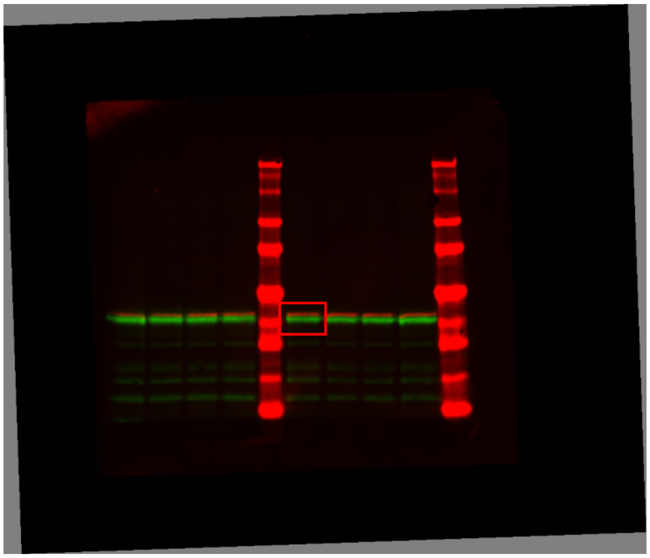

Fig. 10 Day 1 CON

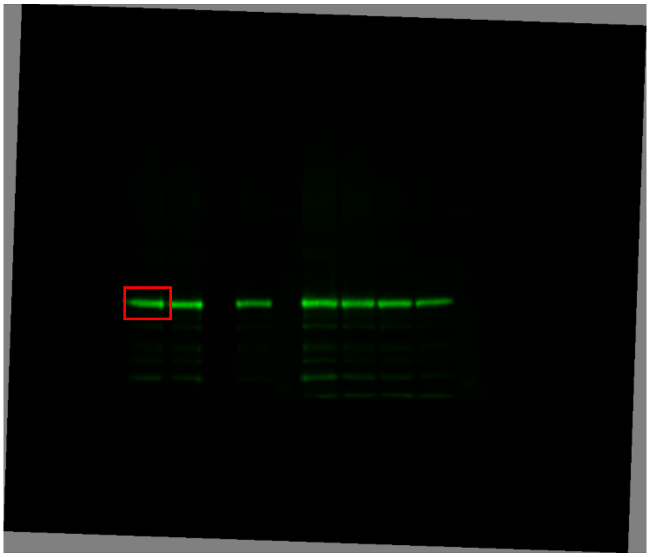

Fig. 10 Day 1 EEAcute

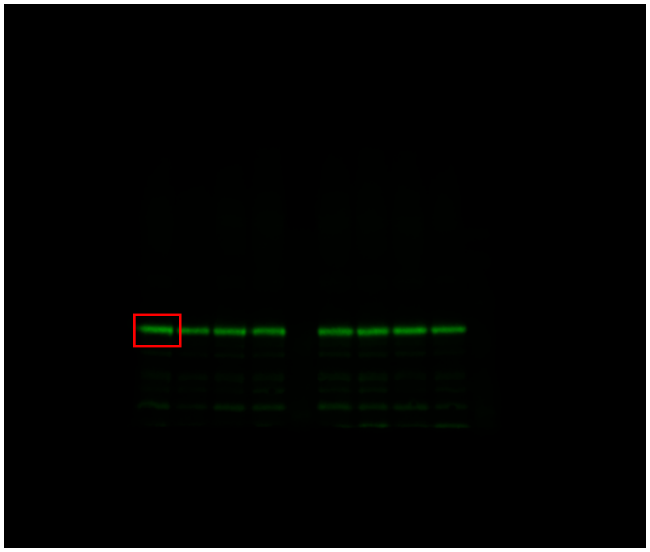

Fig. 10 Day 30 CON

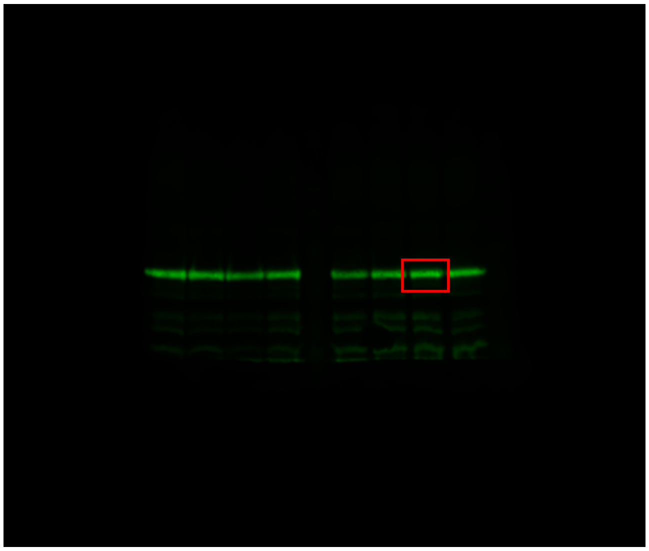

Fig. 10 Day 30 EEAcute

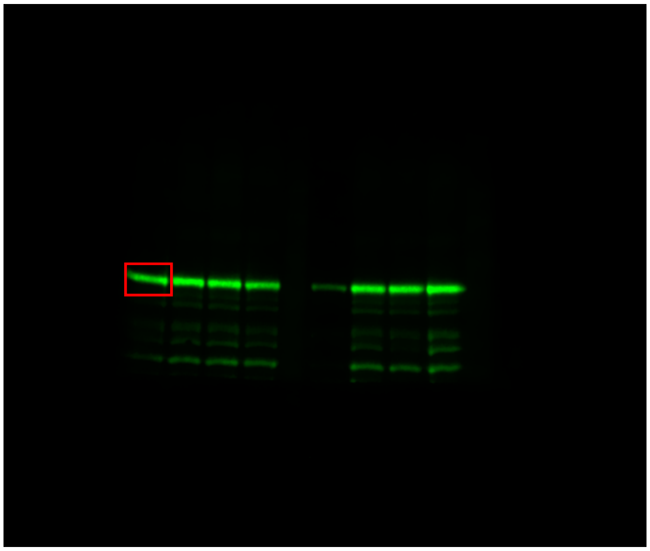

Fig. 10 Day 30 EEChronic

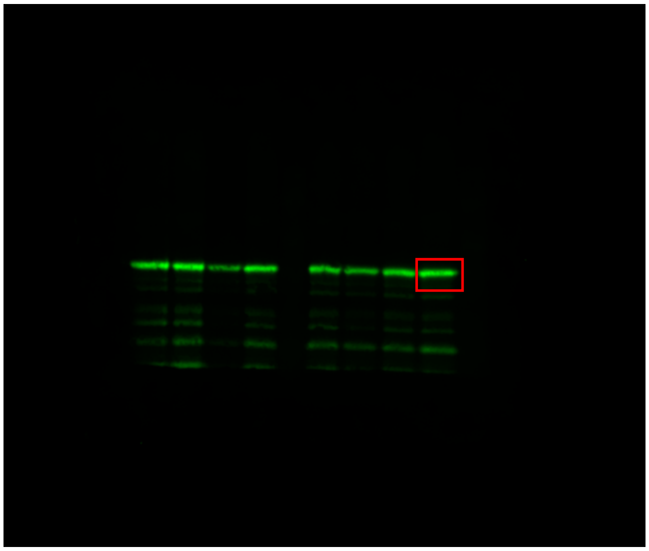

Fig. 11A

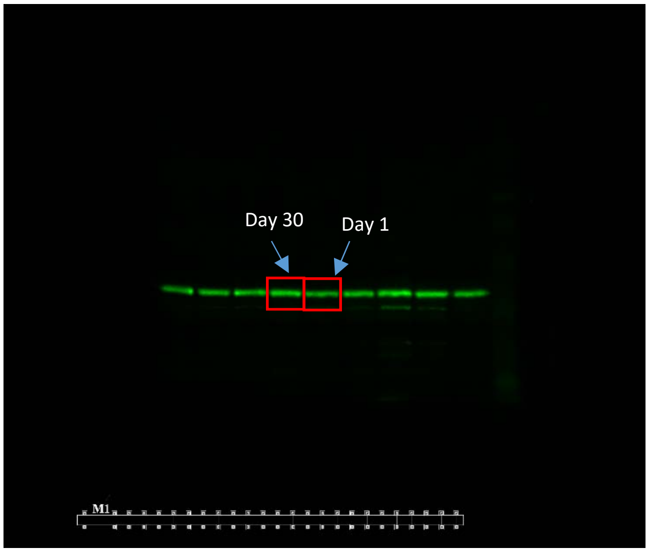

Fig. 11B Day 1

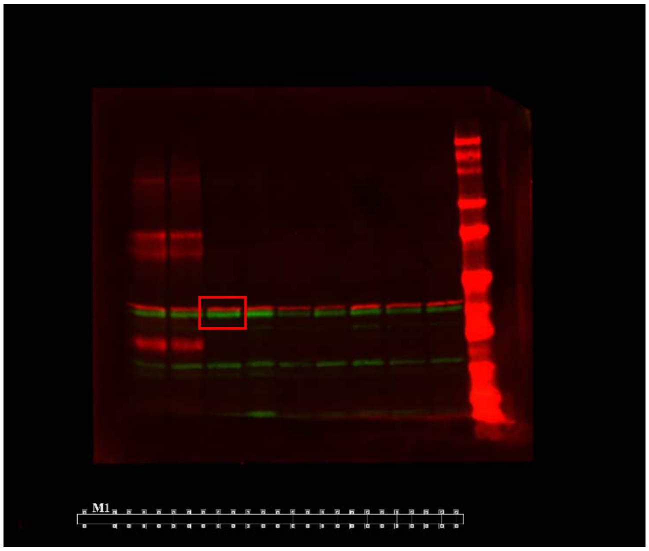

Fig. 11B Day 30

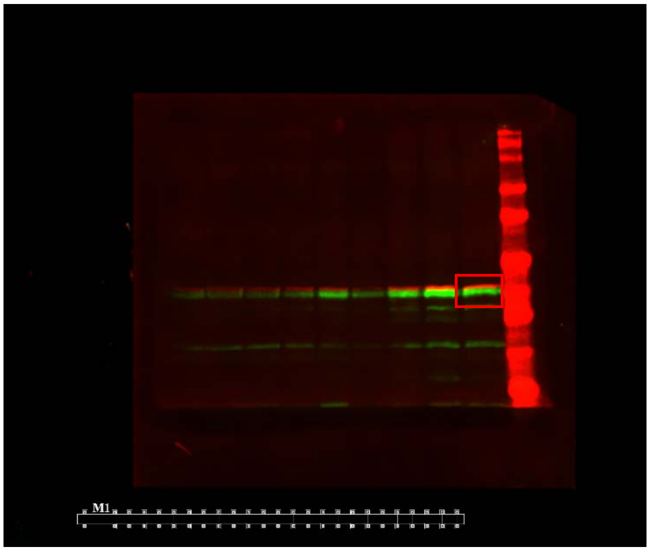

Supplement: Supplementary file 1 — Supplementary Information [file 41598_2018_29625_MOESM1_ESM.pdf]
